# Anticancer Cytotoxic Activity of Bispidine Derivatives Associated with the Increasing Catabolism of Polyamines

**DOI:** 10.3390/molecules27123872

**Published:** 2022-06-16

**Authors:** Ekaterina V. Neborak, Altynay B. Kaldybayeva, Lylia Bey, Aigul Y. Malmakova, Anna S. Tveritinova, Abdullah Hilal, Valentina K. Yu, Maria V. Ploskonos, Marina V. Komarova, Enzo Agostinelli, Dmitry D. Zhdanov

**Affiliations:** 1 Department of Biochemistry, Peoples’ Friendship University of Russia (RUDN University), 8 Miklukho-Maklaya St., Moscow 117198, Russia; beylylia60@gmail.com (L.B.); 1032172705@rudn.ru (A.S.T.); 2 Laboratory of Chemistry of Synthetic and Natural Medicinal Substances, A.B. Bekturov Institute of Chemical Sciences, 59 Tole bi St., Almaty 050000, Kazakhstan; altin_28.94@mail.ru (A.B.K.); malmakova@mail.ru (A.Y.M.); yu_vk@mail.ru (V.K.Y.); 3 Department of Chemistry and Technology of Organic Substances, Natural Compounds and Polymers, Al-Farabi Kazakh National University, 71 al-Farabi Ave, Almaty 050040, Kazakhstan; 4 Laboratory of Medical Biotechnology, Institute of Biomedical Chemistry, 10/8 Pogodinskaya St., Moscow 119121, Russia; hilalabdullahh@gmail.com; 5 Department of Chemistry, Astrakhan State Medical University, Astrakhan 414000, Russia; ploskonoz@mail.ru; 6 Department of Laser and Biotechnical Systems, Samara University, 34 Moskovskoye Shosse, Samara 443086, Russia; marinakom@yandex.ru; 7 Department of Sensory Organs, Faculty of Medicine and Dentistry, Sapienza University of Rome, University Hospital Policlinico Umberto I, I-00161 Rome, Italy; enzo.agostinelli@uniroma1.it; 8 International Polyamines Foundation, ETS-ONLUS, I-00159 Rome, Italy

**Keywords:** bispidines, polyamines, polyamine catabolism, cancer cell lines, antiproliferative activity, screening, polyamine analogs, cytotoxicity, HepG2, WI38, apoptosis induction

## Abstract

Polyamine (PA) catabolism is often reduced in cancer cells. The activation of this metabolic pathway produces cytotoxic substances that might cause apoptosis in cancer cells. Chemical compounds able to restore the level of PA catabolism in tumors could become potential antineoplastic agents. The search for activators of PA catabolism among bicyclononan-9-ones is a promising strategy for drug development. The aim of the study was to evaluate the biological activity of new 3,7-diazabicyclo[3.3.1]nonan-9-one derivatives that have antiproliferative properties by accelerating PA catabolism. Eight bispidine derivatives were synthetized and demonstrated the ability to activate PA catabolism in regenerating rat liver homogenates. However, only three of them demonstrated a potent ability to decrease the viability of cancer cells in the MTT assay. Compounds **4c** and **4e** could induce apoptosis more effectively in cancer HepG2 cells rather than in normal WI-38 fibroblasts. The lead compound **4e** could significantly enhance cancer cell death, but not the death of normal cells if PAs were added to the cell culture media. Thus, the bispidine derivative **4e** 3-(3-methoxypropyl)-7-[3-(1H-piperazin-1-yl)ethyl]-3,7-diazabicyclo[3.3.1]nonane could become a potential anticancer drug substance whose mechanism relies on the induction of PA catabolism in cancer cells.

## 1. Introduction

Polyamines (PAs) are ubiquitous aliphatic polycations (putrescine (Put), spermidine (Spd), and spermine (Spm)) that can interact with biological polyanionic macromolecules [1,2,3,4]. The binding of PA to proteins results in the modulation of the activity of different enzymes [5] and ion channels [6] and supports the functions of cell membranes. Due to their ability to interact with nucleic acids, PAs can influence gene transcription [7,8] and mRNA translation [9,10]. Thus, PAs are essential for different cellular functions, including cell growth and proliferation [11]. In addition to regulating functions in normal cells, they can also play a crucial role in malignant transformation and tumor cell proliferation, as their levels are often elevated in tumor tissues [12]. This elevation can occur due to an imbalance in PA synthesis and degradation that is often observed in cancer tissues in comparison with normal tissues. The expression of enzymes responsible for PA biosynthesis, namely, S-adenosylmethionine decarboxylase and/or ornithine decarboxylase, is increased in gastric cancers [12], breast cancers [13], neuroblastoma [14,15], prostate cancers, leukemias and other types of cancer [16]. The inhibition of these enzymes resulted in tumor sensitization to anticancer therapy [17,18] or in a decrease in tumor aggressiveness [19]. The degradation pathway of PAs includes different di- and polyamine oxidases [2,3] that are often reduced in cancer tissues [20,21], while the catabolic products of PA oxidation are able to induce apoptosis in normal and tumor cells [22,23,24,25]. Thus, activation of the PA catabolic pathway, primarily through a direct interaction with PA oxidases, may be a promising strategy for cancer therapy [26,27,28]. The aim of the current study was to search for a potential anticancer agent among the activators of PA catabolism.

Bispidines (diazabicyclononanes) are well known in medicinal chemistry, providing a wide variety of biological activities. They are intensively studied as anticancer [29] or antiviral [30] agents as well as suitable ligands for radiopharmaceuticals [31] and theranostic drugs [32]. Our previous study showed that bicyclononane derivatives were among the most potent activators of the PA catabolic pathway [33]. In the present study, a set of previously described diazabicyclononan-9-one derivatives [34,35,36] and novel synthesized diazabicyclononan-9-one derivatives has been tested for their ability to activate the PA-degrading pathway and to induce cancer cell death through such activation. We found an enhancement of PA catabolism in regenerating rat liver homogenates and increased PA-dependent cytotoxicity for human cancer cells for some bispidine derivatives.

## 2. Results

### 2.1. Synthesis of 3,7-Diazabicyclo[3.3.1]nonane Derivatives

**Figure 1:**
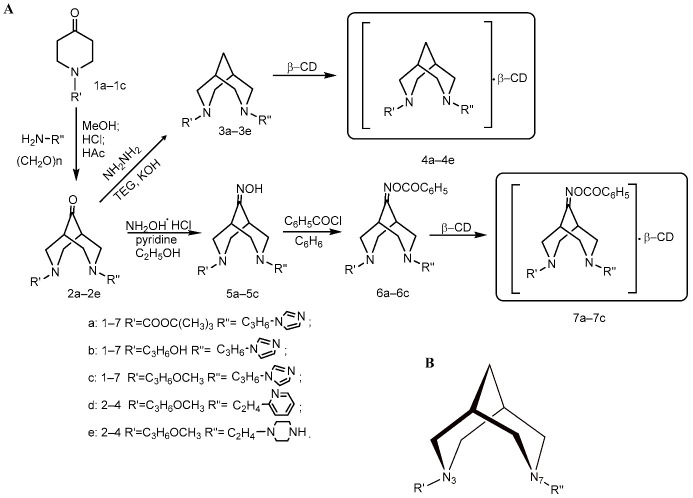
Scheme of the synthesis of 3,7-diazabicyclo[3.3.1]nonane derivatives (**A**) and the general structure of the synthesized compounds (**B**).

The synthesized 3,7-diazabicyclo[3.3.1]nonan-9-ones (bispidine derivatives) were tested as potential modulators of PA metabolism and anticancer agents. They were prepared in the Laboratory of Chemistry of Synthetic and Natural Medicinal Compounds of A.B. Bekturov Institute of Chemical Sciences. The scheme in Figure 1 illustrates the syntheses and the variety of bicyclo[3.3.1]nonane and O-benzoyloxime complexes prepared with β-cyclodextrin. Compounds **3a**–**3e** and **6a**–**6c** readily formed complexes **4a**–**4e** and **7a**–**7c** with β-cyclodextrin, respectively.

3,7-Diazabicyclo[3.3.1]nonan-9-ones (**2a**–**2e**) were synthesized (Figure 1A) by simultaneous Mannich condensation of 1-Boc-, 1-(3-hydroxypropyl)- and 1-(3-methoxypropyl)-piperidin-4-ones (**1a**–**1c**) with paraformaldehyde and the primary amines 1-(3-aminopropyl)imidazole, 1-(2-aminoethyl)pyridine and 1-(2-aminoethyl)piperazine in an acetic-methanol medium.

The corresponding 3,7-diazabicyclo[3.3.1]nonanes (**3a**–**3e**) were obtained by the Kizhner-Wolf reaction, reducing compounds **2a**–**2e** under the action of hydrazine hydrate in the presence of KOH in triethylene glycol.

Prolonged reflux of bicyclic ketones **2a**–**2e** with hydroxylamine hydrochloride led to the corresponding oximes **5a**–**5c**, the subsequent acylation of which by benzoyl chloride synthesized O-benzoyloximes **6a**–**6c**.

Column chromatography (on III activity alumina, the eluent is benzene:dioxane 5:1) was used for purification of novel bicyclic ketones, nonanes, bicyclic oximes and O-benzoyl oximes. The completion of the reactions was monitored by TLC. 1H and 13C NMR spectroscopies were used to determine the structures of the synthesized substances.

In the IR spectrum of 3,7-diazabicyclo[3.3.1]nonan-9-ones (**2a**–**2e**), characteristic bands of stretching vibrations of the carbonyl group appear.

The formation of bicyclic amines was evidenced by the absence of the absorption band of the carbonyl group in the IR spectra of compounds **3a**–**3e**.

When comparing the 13C NMR spectra of 3,7-diazabicyclo[3.3.1]nonanes (**3a**–**3e**) with the spectra of the starting bicyclic ketones (**2a**–**2e**), it can be seen that they lack the signal of the carbon atom characteristic of the carbonyl group, while in the upfield part of the spectrum, a triplet signal of the carbon atom of the methylene group appears in the 9th position.

The absorption bands of the C=N bond and the OH group are identified in the IR spectra of oximes (**5a**–**5c**). The most intense band in **6a**–**6c** corresponds to the benzoyloxy-carbonyl group. The 13C NMR spectra of oximes (**5a**–**5c**) and their esters (**6a**–**6c**) showed resonances for C-9. In the 1H NMR spectra, H1,5 protons were observed as separate signals. The weakest signal was assigned to the proton of the NOH group (**5a**–**5c**). Signals for the protons of the phenyl ring were observed in the spectrum of the O-benzoyloxime derivatives (**6a**–**6c**).

To study the biological properties of the novel derivatives of 3,7-diazabicyclo[3.3.1]nonan-9-ones, their water soluble complexes with β-cyclodextrin (**4a**–**4e**, **7a**–**7c**) were synthesized. The complexes melted above 240 °C with decomposition.

3,7-Diazabicyclo[3.3.1]nonan-9-one derivatives (**1c**, **2a**, **2c**, **2d**, **3a**, **3c**, **3d**, **4a**, **4c**, **4d**) have been studied for myelo-stimulating and plant growth-regulating activity [34,35,36]. The physicochemical properties of novel derivatives **2b**, **2e**, **3b**, **3e**, **4b**, **4e**, and **7a**–**7c** are described in the chemical experimental section.

The core bispidine structure of 3,7-diazabicyclo[3.3.1]nonane contains two heterocyclic nitrogens—N3 and N7 (Figure 1B). Different combinations of side N3- and N7-radicals provide a set of five compounds **4a**–**4e**, three of which were supplied with benzoyloxim partners **7a**–**7c** and were also included in the investigation.

### 2.2. The Activation of Polyamine Catabolism in Regenerating Rat Liver Homogenates

**Figure 2:**
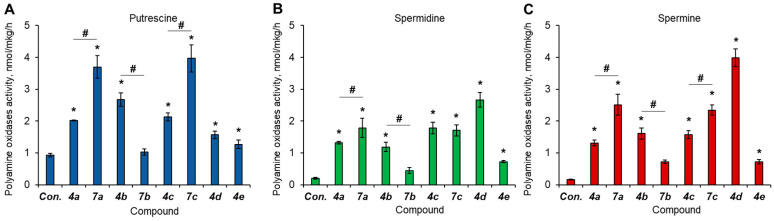
The influence of the tested compounds on the rate of PA oxidation. Rat liver homogenates were incubated with 20 µM compounds, and polyamine oxidase activity was determined for (**A**) Put, (**B**) Spd and (**C**) Spm as described in the experimental section. *N* = 4. * *p* ≤ 0.05 vs. control. # *p* ≤ 0.05 between bicyclononane and its benzoyloxym-free partner. Con., control.

Eight synthetized bispidine derivatives were tested for their ability to activate oxidation of PAs in the homogenates of regenerating rat liver. Put is normally oxidized by diamine oxidase, which is a copper-containing enzyme that produces ammonia and H_2_O_2_ [37]. Spd and Spm are substrates of Spd/Spm-acetyltransferase and acetyl-polyamine oxidase. The results demonstrate that all the compounds could activate the oxidation of all three PAs (Put, Spd, Spm) at varying rates (Figure 2).

Benzoyloxym compounds might be considered prodrugs with partly blocked activity due to the presence of this specific group that might be eliminated by hepatic enzymes. Our set of bispidine derivatives includes three such “drug–prodrug” pairs: **4a**/**7a**, **4b**/**7b**, and **4c**/**7c**. Pairs **4a**/**7a** and **4c**/**7c** contain a hydrophobic fragment at N3, while the **4b**/**7b** pair contains a terminal OH group at N3 that provides some hydrophilicity to this fragment. The general tendency for oxidation of different PAs against the background of the action of the compounds is the following: only pairs **4b**/**7b** demonstrated true drug–prodrug-like activity, as the accelerating effect on PA catabolism was significantly lowered by benzoyloxymation. Compounds **7a** and **7c**, by contrast, were more active than their benzoyloxym-free partners **4a** and **4c**, respectively, which demonstrates that the benzoyloxym moiety might enhance the activatory action of these diazabicyclononanes toward PA oxidation. Compounds **4d** and **4e** are both benzoyloxym-free but differ by the side radical at N3, namely, **4d** contains an aryl moiety, while **4e** contains an aliphatic heterocycle. The comparison of these two compounds reveals that the aromatic radical provides stronger PA catabolism activatory properties to the molecule as the PA oxidation becomes 3–5-fold higher under the action of **4d** than that of **4e**. Thus, **4d** becomes a leader among all the tested compounds in regard to the activation of Spm oxidation.

### 2.3. Toxicity of Bicyclononane Derivatives toward Cancer Cells

**Figure 3:**
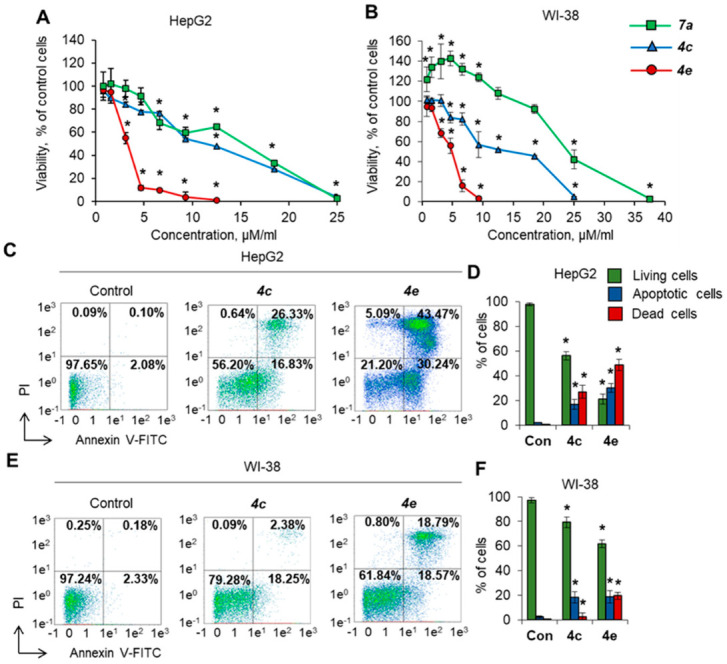
Cell viability in the presence of diazabicyclononane derivatives. Cancer cells and normal fibroblasts were cultivated for 72 h in the presence of different concentrations of **4c**, **4e** and **7a**. Cell viability measured by MTT test for (**A**) HepG2 and (**B**) WI-38 cell lines. (**C**,**E**) Representative flow cytometry diagrams for cells incubated with 25 µM **4c** or **4e** and labeled with Annexin V-FITC and PI demonstrating the induction of apoptosis. The ratio of living cells (low left quadrants), apoptotic cells (low right quadrants), dead necrotic cells (upper left quadrant) and dead apoptotic cells (upper right quadrant) are presented. (**D**,**F**) Histograms of live, apoptotic and dead cells incubated with **4c** or **4e**. Con, control untreated cells. PI, propidium iodide. *N* = 8 for MTT assay. *N* = 4 for the flow cytometry study. * *p* ≤ 0.05 vs. control untreated cells.

To test the cytotoxic activity (loss of cell viability) of the compounds, HepG2 human liver carcinoma cells and WI-38 normal fibroblasts were cultivated in the presence of different concentrations of synthesized compounds, and cell viability and apoptosis induction were measured after 72 h of incubation. Table 1 represents IC50 values toward the chosen cell lines. Only three compounds (**4c**, **4e**, **7a**) demonstrated selectivity toward cancer HepG2 cells (Figure 3A) with significantly less cytotoxicity and higher IC50 toward normal cells (Figure 3B). Compound **4e** was the most active and could induce a gradual decrease in HepG2 cell viability in the range of concentrations of 3–9 µM. Almost all cells were not viable after incubation at higher concentrations. Compound **7a** was less active and could induce a gradual decrease in cell viability at concentrations of 7–25 µM. Compound **4c** demonstrated moderate activity and gradually suppressed cell growth at concentrations of 3–25 µM. Compound **7a** demonstrated the ability to increase the viability of normal WI-38 fibroblasts at low concentrations of 1–9 µM and was therefore excluded from further studies.

To investigate the type of cell death, cells were incubated with 25 µM **4c** or **4e**, and the induction of apoptosis was assessed by labeling cell membrane phosphatidylserine with annexin V-FITC and cell DNA with PI combined with flow cytometry. The results of the apoptosis evaluation were in good agreement with the results from the MTT test. Both compounds induced apoptosis more efficiently in cancer HepG2 cells than in normal WI-38 cells: 56% and 20% of cells remained alive after incubation with **4c** or **4e**, respectively (Figure 3C,D). The tested compound **4c** did not induce apoptosis in normal fibroblasts at a concentration of 25 µM, whereas cells were more sensitive to **4e** (Figure 3E,F). Thus, **4e** demonstrated cytotoxicity against both normal and cancer cells. Taken together, the results demonstrate that the bispidine derivatives **4c** and **4e** possess strong cytotoxic activity and can induce apoptosis in cancer cells, while normal cells are less sensitive to their action.

### 2.4. Polyamines Enhance the Toxicity of Diazabicyclononane Derivative **4e** toward Cancer Cells

The study of cell viability and apoptosis induction demonstrated that compound **4e** could induce a higher rate of cancer cell death than **4c**, although no correlation was revealed between the ability to enhance PA catabolism (Figure 2) and cell viability or apoptosis induction (Figure 3) of the tested compounds. Therefore, subsequent experiments were performed with the most active compounds, **4c** and **4e**.

The degradation of PAs is commonly reduced and abnormal in cancer cells [20,21], while the products of PA oxidation are known to be able to induce cancer cell death by apoptosis [40,41]. To determine whether the cytotoxic activity of diazabicyclononane derivatives is truly associated with the activation of PA catabolism, we incubated HepG2 or WI-38 cells with a maximum nontoxic concentration (MNTC) of 2 µM for compounds **4c** and **4e** in the presence of 1 or 10 µM Spm or Spd. The controls were incubated with **4c** and **4e** and with each of PAs separately.

**Figure 4:**
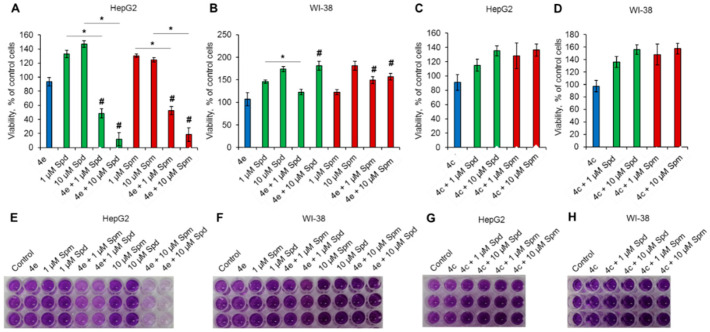
Cytotoxic activity toward cancer cells of diazabicyclononane derivative **4e** but not **4с** depends on PA presence in growth media. Cancer cells and normal fibroblasts were cultivated for 72 h in the presence of MNTC of **4c** or **4e**. Cell viability measured by MTT test for (**A**,**C**) HepG2 and (**B**,**D**) WI-38 cell lines in the presence of 1 or 10 µM Spd or Spm. (**E**–**H**) Representative pictures of the MTT test for cells incubated with diazabicyclononane derivative and PAs. *N* = 8. # *p* ≤ 0.05 vs. cells treated with diazabicyclonane derivative **4c** or **4e**. * *p* ≤ 0.05 between groups of cells.

Both PAs in a single compound treatment promoted the viability of HepG2 and WI-38 cells in the MTT test (Figure 4A,B,E,F). The combination of nontoxic concentrations of **4e** and **4c** with PAs resulted in different effects on cell viability. Namely, the **4e** + 1 µM Spd/Spm combination caused 50% cell death in HepG2 cells (Figure 4A,E). The combination of **4e** + 10 µM Spd/Spm demonstrated even greater cytotoxicity, leaving only 10–20% of living cells. This effect suggests a PA-dose-dependent enhancement of the cytotoxicity of **4e**. In contrast, Spd/Spm-induced WI-38 proliferation was slightly lowered by coincubation with **4e** (Figure 4B,F). Compound **4c** was not able to induce any significant reduction in cell viability in the presence of PAs (Figure 4C,D,G,H). Moreover, **4c** did not affect the viability of either cancer or normal cells in PA-containing media. These results suggest that the cytotoxic activity of **4c**, which is shown in Figure 3, is not associated with the catabolism of PAs, and the addition of PAs to **4c**-treated cells did affect cell viability.

**Figure 5:**
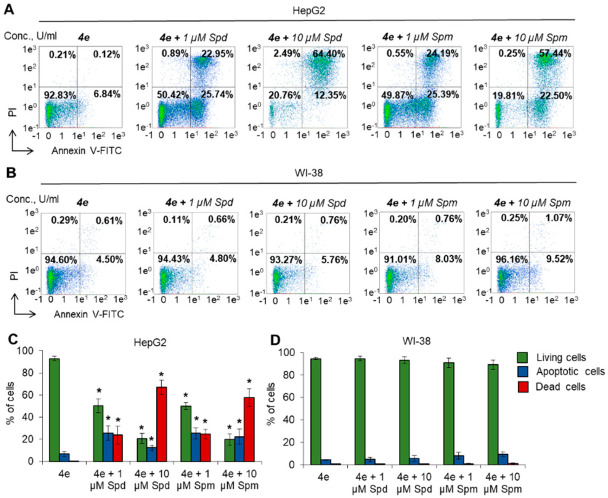
Induction of apoptosis in cancer cells by **4e** in the presence of PAs. Cells were incubated with **4e** MNTC and 1 or 10 µM Spd or Spm, and the induction of apoptosis was detected by flow cytometry (**A**,**B**). Representative flow cytometry diagrams for cells labeled with Annexin V-FITC and PI. The ratio of living cells (low left quadrants), early apoptotic cells (low right quadrants), dead necrotic (upper left quadrant) and dead apoptotic (upper right quadrant) cells are presented. Histograms of live, early apoptotic and dead cells incubated with **4e** and each PA vs. cells incubated with **4e** (**C**,**D**). *N* = 4. * *p* ≤ 0.05.

To reveal the type of cell death induced by the combination of **4e** and PAs, cells were labeled with Annexin-V and FITC and subjected to flow cytometry. The results were in good agreement with the MTT test. Significant induction of HepG2 cell apoptosis was observed after incubation with **4e** in the presence of each PA (Figure 5A,C). Higher concentrations of Spd or Spm could induce a greater degree of cell death through apoptosis. However, incubation of normal WI-38 cells with PAs did not have any significant influence on cell death (Figure 5B,D). The results of this experiment demonstrate that diazabicyclononane derivative **4e** is able to induce apoptosis at the highest nontoxic concentration in the presence of PAs in HepG2 cancer cells but not in normal WI-38 fibroblasts.

## 3. Discussion

Our study revealed biological activity among novel bispidine derivatives. The tested compounds can activate PA catabolism, and two of them, **4e** and **4c**, demonstrate pronounced toxic activity toward cancer cells. Our experiment also revealed a PA-dependent enhancement of the cytotoxicity of the most active bispidine derivative **4e**. The results of our study demonstrate that tumor selectivity and potent anticancer activity for this compound could be achieved in combination with PAs.

Our study is in accordance with the recent work by Kanamori et al. [42], who demonstrated that exogenous bovine serum amine oxidase and PA could induce apoptosis in cancer but not normal cells. However, we aimed to enhance the same PA-degrading pathway using small molecule drug substances to activate endogenous enzymes of PAs catabolism.

Previously, it was shown that exogenous PAs can promote tumor and normal cell proliferation [43] and provide a cytoprotective action for normal fibroblasts [44]. In accordance, we demonstrated the promotion of WI-38 and HepG2 viability after cell incubation with PAs (Figure 4A,B). The viability of normal WI-38 cells in the presence of combinations **4e** + Spd/Spm and **4c** + Spd/Spm was not significantly different from that induced by PAs alone. These results suggest that the tested bispidine derivatives had no effect on the viability of normal cells. In case of cancer HepG2 cells, these two compounds demonstrated opposite effects: **4c**, which was not active at MNTC, caused no cell death when added in combination with PAs, while **4e** cytotoxic activity was potentiated by exogenous PAs.

The products of PA catabolism (mainly reactive oxygen species (ROS), such as hydrogen peroxide and acrolein) are capable of inducing cell death via apoptosis [25,26,32,33]. Apoptosis was the type of cell death in HepG2 cells when **4e** was coincubated with Spd/Spm. The absence of cell death at the MNTC of the compound suggests that the initial cellular content of PAs in tumor HepG2 cells (Table 2) is not enough high to produce sufficient amounts of toxic metabolites of PA decomposition to cause apoptosis. We believe that the addition of exogenous PAs resulted in their oxidation by bispidine-activated PA catabolic enzymes and the generation of cytotoxic products that could trigger cell death. Such an effect was specific only for **4e** but not for **4c**, despite its more pronounced ability to accelerate PA catabolism shown in regenerating rat liver homogenates.

**Figure 6:**
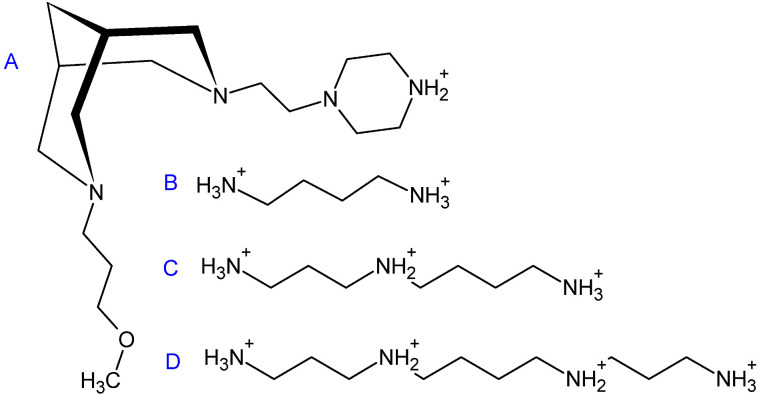
Comparison of the structure of **4e** with the positively charged side of piperazin and the structures of PAs. (**A**)—the most active compound **4e**: 3-(3-methoxypropyl)-7-[3-(1H-piperazin-1-yl)ethyl]-3,7-diazabicyclo[3.3.1]nonane. (**B**)—Put, (**C**)—Spd, (**D**)—Spm.

We believe that the low transmembrane penetration or intracellular metabolism of **4c** may be the reason for its low activity in living cells. The tested compounds were converted into complexes with β-cyclodextrins that are widely used in medicinal chemistry to improve water solubility and the transportation of hydrophobic drugs to the target cell membrane barriers. The penetration of such complexes inside the cell may occur via two main pathways [45]. The first is endo- and/or pinocytosis, which is typical for similar complexes regardless of the chemical composition. The second mechanism relies on the dissociation of complexes with the release of active substances that can pass through the cell membrane. This pathway depends on the physical and chemical properties of the active compound. The mechanism of cellular uptake of bispidines has not yet been reported. Compound **4e** has some structural features that distinguish it from other compounds and may potentiate its activity. In particular, it contains nonaromatic piperazinyl-ethyl-side radicals at position N7, while all other tested substances include aromatic radicals at that position. This saturated diazacyclic fragment has a secondary amino group (Figure 6), is protonated and positively charged under physiological conditions due to the basic properties of piperazine, which are much stronger than those in imidazoles or pyridines [46]. Moreover, the positive charge is not delocalized in aliphatic piperazine ring unlike aromatic imidazole or pyridine rings. The saturation of the C-C bonds and positively charged nitrogen make **4e** structurally more similar to natural aliphatic PAs (Figure 6). This structural similarity may promote the interaction of **4e** with natural PA transporters [47] and their penetration through the membrane after the dissociation of the bispidine-β-cyclodextrin complex. Thus, we can assume that the piperazine moiety unique to the lead compound **4e** may facilitate its uptake by the cells, thus enhancing its intracellular toxic effect.

Another interesting observation is the selectivity of **4e** toward the cancer cell line HepG2, which is obvious and needs to be explained. The concentrations of PAs in HepG2 and WI-38 cells are comparable (Table 2), according to the literature data, and are much lower than the amount of exogenously added PA in our experiments. Thus, they cannot be responsible for the different effects of **4e** on cell viability in these two cell lines. We suppose that the differences in PA metabolism enzyme activities in cancer and normal cells are more likely to be the reason for the observed selective cytotoxicity of **4e**. The available data on PA metabolism characteristics in the experimental cell lines are summarized in Table 2. Additionally, normal hepatocytes and mesenchymal fibroblasts have been added for a better illustration, as not all the parameters for the experimental cell lines have been found in the literature. The absolutely zero transcription level of one PA catabolic enzyme, acetl-polyamine oxidase (APAO), in HepG2 cells was reported in earlier studies. This feature is one of the key differences between normal and tumor tissue metabolism [2,3,20,21,22,23,24,25]. In contrast, spermine oxidase (SMOX) is more active in cancer HepG2 cells than in normal cells. The overexpression of this PA catabolic enzyme is often associated with cellular malignization [37] due to a double-edged role of ROS in tumor progression. Our compounds are potent activators of the PA catabolic pathway, and a rapid ROS storm may cause apoptosis rather than malignization and tumor promotion. In addition to direct activation, induction of the corresponding gene expression might also take place, although this hypothesis needs to be further verified.

The benzoylated and nonbenzoylated pairs did not demonstrate any pronounced drug/prodrug activity. Instead, adding benzoyloxim to the basic structures provided mostly greater activity toward PA catabolism and antiproliferative action.

As demonstrated in Figure 4, adding each of PAs as a substrate to boost toxicity provided convincing evidence for the potential of bispidines. Minimally toxic bispidines strongly synergized the potency of the two PAs in the tumor cell line but not in fibroblasts. Taken together, the results of the study demonstrate that synthesized bispidine derivatives can induce cancer cell death and the activation of PA catabolism. In the present study, we showed for the first time that the bispidine derivative **4e** 3-(3-methoxypropyl)-7-[3-(1H-piperazin-1-yl)ethyl]-3,7-diazabicyclo[3.3.1]nonane can be subjected to PA-based drug development.

## 4. Materials and Methods

### 4.1. Chemical Experimental Part

#### 4.1.1. Reagents and Equipment

Primary amines, paraform and 1-Boc-piperidin-4-one (**1a**) were purchased from Aldrich (Louis Street, MO, USA). IR spectra were recorded on a Nicolet 5700 instrument between KBr plates. 1H and 13C NMR spectra were recorded on a JNM-ECA Jeol 400 spectrometer (frequencies 399.78 and 100.53 MHz, respectively) using DMSO-d6 solvent. The elemental analysis data were consistent with the calculated values. Column chromatography and thin-layer chromatography were carried out on alumina (Al_2_O_3_) of the third degree of activity, and Rf compounds were given for this type of plate. The spots were developed in iodine vapors. The IR and NMR spectra for the synthesized compounds are presented in the Supplementary File.

#### 4.1.2. Syntheses of Bispidine-9-Ones (Compounds **2a**–**2e**)

*1-(3-Hydroxypropyl)piperidin-4-one(***1b**). A total of 385 mL of absolute toluene and 16.26 g (0.707 mol) of metallic Na were placed in a three-necked flask equipped with a mechanical stirrer, reflux condenser with a calcium chloride tube, and dropping funnel. The reaction mixture was heated to 110 °C in an oil bath until the sodium dissolved (to obtain suspensions of sodium in toluene) for 25–30 min. Then, the reaction mixture was cooled to 75–80 °C, then 128 mL of methanol was added dropwise and it was stirred at 75 °C for 2 h. Afterwards, a straight condenser was attached to the flask, and the reaction mixture was heated. A solution of 174.61 g (0.707 mol) of diester was added using a dropping funnel at 110 °C, while the azeotropic mixture of toluene and methanol was simultaneously distilled off by direct distillation. The rate of dropping of the mixture of diester and methyl alcohol should be equal to the rate of distillation of the solvents. When the temperature reached 110 °C, becoming equal to the boiling point of toluene, the heating was stopped. While cooling (ice was used for cooling) and stirring, a solution of 301 mL of concentrated hydrochloric acid and 301 mL of distilled water was gradually added. The formed organic and aqueous layers were separated. The lower aqueous acidic layer was boiled at 100 °C for 8 h. The progress of the reaction was monitored using a solution of 1% FeCl_3_. The color changed to maroon. The solution was alkalized with NaOH to pH 12, extracted with chloroform, and dried over anhydrous MgSO_4_. The solvent was evaporated, and the residue was purified by column chromatography on Al_2_O_3_, benzene:dioxane = 5:1. A total of 21.04 g (19%) of 1-(3-hydroxypropyl)piperidin-4-one (**1b**) was obtained in the form of light yellow oil with R_f_ = 0.65 (Al_2_O_3_, benzene:isopropanol = 7:1).

Found, %: C 61.11, H 9.58, N 8.90. C_8_H_15_NO_2_.

Calculated, %: C 61.14, H 9.55, N 8.92.

IR spectrum, cm^−1^: 1716 (ν_C=__О_).

^1^H NMR spectrum, δ, ppm: *bicyclononan-9-one:* 1.56 (2H, Н-1_e__q_,5_e__q_), 2.07-2.45 (2H, Н-6_ax_,8_ax_), 2.85-3.22 (2H, Н-2_ax_,4_ax_), 3.47–3.59 (2H, Н-6_e__q_,8 _e__q_), 3.81–3.89 (2H, Н-2 _e__q_,4 _e__q_). *hydroxypropyl:* 4.60 (1H, NCH_2_CH_2_CH_2_OH).

^13^C NMR spectrum ppm: *bicyclononan-9-one*: 59.2 (C-1,5), 60.2 (C-2,4,6,8), 214.5 (C-9). *hydroxypropyl:* 27.5 (NCH_2_CH_2_CH_2_OH), 49.7, 63.7 (NCH_2_CH_2_CH_2_OH).

*1-Methoxypropyl-piperidin-4-one* (**1c**). The procedure for the synthesis of 1-methoxypropyl-piperidin-4-one (**1c**) was described earlier [34].

*3-Boc-7-[3-(1H-imidazol-1-yl)propyl]-3,7-diazabicyclo[3.3.1]nonan-9-one* (**2a**). The procedure for the synthesis of 3-Boc-7-[3-(1H-imidazol-1-yl)propyl]-3,7-diazabicyclo[3.3.1]nonan-9-one (**2a**) was described earlier [35].

*3-(3-Hydroxypropyl)-7-[3-(1H-imidazol-1-yl)propyl]-3,7-diazabicyclo[3.3.1]nonan-9-one (***2b***).* In a three-necked flask equipped with a stirrer, reflux condenser and dropping funnel, 40 mL of methanol was deoxygenated under a stream of nitrogen. After 30 min, a mixture of 9 mL (0.076 mol) of 1-(3-aminopropyl)imidazole), 9.17 g (0.304 mol) of paraform, 4 mL of concentrated hydrochloric acid, and 6 mL of glacial acetic acid was added and stirred for 15 min in an atmosphere of nitrogen. A solution of 12 g (0.076 mol) of 1-(3-hydroxypropyl)piperidin-4-one (**1b**) and 6 mL of glacial acetic acid in 43 mL of methanol was added dropwise. After 10 h of heating the reaction mixture at 60–65 °C, a second equivalent of paraform was added and held for another 10 h at the same temperature. The solvent was evaporated, and the residue was dissolved in 121 mL of water. The extraction of neutral products was carried out with diethyl ether. The aqueous layer was alkalinized to pH 12, and the organic part was extracted with chloroform and dried over MgSO_4_. The solvent was evaporated, and the obtained product was purified by column chromatography on Al_2_O_3_:benzene:dioxane 5:1. A total of 13.84 g (59%) of 3-(3-hydroxypropyl)-7-[3-(*1H-*imidazol-1-yl)propyl]-3,7-diazabicyclo[3.3.1]nonan-9-one (**2b**) was obtained in the form of a light yellow oil with R_f_ = 0.41 (Al_2_O_3_, benzene:isopropanol = 6:1).

Found, %: C 62.72, H 8.52, N 18.27. C_16_H_26_N_4_O_2_.

Calculated, %: C 62.75, H 8.50, N 18.30.

IR spectrum, cm^−1^: 1729 (ν_C=__О_), 1071 (ν_C-__О-__C_).

^1^H NMR spectrum, δ, ppm: *bicyclononan-9-one:* 1.54 (2 H, Н-1_e__q_,5_e__q_), 2.07–2.45 (2 H, Н-6_ax_,8_ax_), 2.85–3.22 (2 H, Н-2_ax_,4_ax_), 3.47–3.59 (2 H, Н-6_e__q_,8 _e__q_), 3.81–3.89 (2 H, Н-2 _e__q_,4 _e__q_). *1H-imidazol-1-yl)propyl:* 6.75–6.78, 6.82, 7.33 (3H, CH_imidazol_). Side chains: 1.54 (2H, NCH_2_CH_2_CH_2_N); 1.69–1.81 (2H, NCH_2_CH_2_CH_2_OH*). hydroxypropyl:* 4.60 (1H, NCH_2_CH_2_CH_2_OH).

^13^C NMR spectrum ppm: *bicyclononan-9-one*: 59.4 (C-1,5), 61.4 (C-2,4,6,8), 214.5 (C-9). *(1H-imidazol-1-yl)propyl:* 119.0 (CH_imidazol_), 128.7, 137.2. Side chains: 29.3 (CH_2_CH_2_CH_2_), 44.9, 47.4 (CH_2_CH_2_CH_2_). *hydroxypropyl:* 27.5 (NCH_2_CH_2_CH_2_OH), 49.7, 63.7 (NCH_2_CH_2_CH_2_OH).

*3-(3-Methoxypropyl)-7-[3-(1H-imidazol-1-yl)propyl]-3,7-diazabicyclo[3.3.1]nonan-9-one* (**2c**). The procedure for the synthesis of 3-(3-methoxypropyl)-7-[3-(1H-imidazol-1-yl)propyl]-3,7-diazabicyclo[3.3.1]nonan-9-one (**2c**) was described earlier [34].

*3-(3-methoxypropyl)-7-[2-(pyridin-2-yl)ethyl]-3,7-diazabicyclo[3.3.1]nonan-9-one* (**2d**). The procedure for the synthesis of 3-(3-methoxypropyl)-7-[2-(pyridin-2-yl)ethyl]-3,7-diazabicyclo[3.3.1]nonan-9-one (**2d**) was described earlier [36].

*3-(3-Methoxypropyl)-7-[2-(piperazin-1-yl)]-3,7-diazabicyclo[3.3.1]nonan-9-one* (**2e**). In a three-necked flask equipped with a stirrer, reflux condenser and dropping funnel, 50 mL of methanol was deoxygenated under a stream of nitrogen. After 30 min, a mixture of 7.55 g (0.058 mol) of 1-(2-aminoethyl)piperazine, 7 g (0.304 mol) of paraform, 3 mL of concentrated hydrochloric acid, and 4 mL of glacial acetic acid was added and stirred for 15 min in an atmosphere of nitrogen. A solution of 10 g (0.058 mol) of 1-(3-methoxypropyl)piperidin-4-one (**1c**) and 4 mL of glacial acetic acid in 43 mL of methanol was added dropwise. After 10 h of heating the reaction mixture at 60–65 °C, a second equivalent of paraform was added and held for another 10 h at the same temperature. The solvent was evaporated, and the residue was dissolved in 119 mL of water. The extraction of neutral products was carried out with diethyl ether. The aqueous layer was alkalinized to pH 12, and the organic part was extracted with chloroform and dried over MgSO_4_. The solvent was evaporated, and the obtained product was purified by column chromatography on Al_2_O_3_:benzene:dioxane 5:1. A total of 17.15 g (67%) of 3-(3-methoxypropyl)-7-[2-(piperazin-1-yl)ethyl]-3,7-diazabicyclo[3.3.1]nonan-9-one (**2e**) was obtained in the form of a light yellow oil with R_f_ = 0.32 (Al_2_O_3_, benzene:isopropanol = 6:1).

Found, %: C 62.89, H 9.81, N 19.79. C_17_H_32_N_4_O_2_.

Calculated, %: C 62.96, H 9.88, N 19.75.

IR spectrum, cm^−1^: 1732 (ν_C=__О_), 1089 (ν_C-__О-__C_).

^1^H NMR spectrum, δ, ppm: *bicyclononan-9-one*: 1.55 (2H, Н-1_e__q_,5_e__q_), 2.80–2.96 (2H, Н-6_ax_,8_ax_), 2.98 (2H, Н-2_ax_,4_ax_), 3.47–3.59 (2 H, Н-6_e__q_,8 _e__q_), 3.87 (2H, Н-2 _e__q_,4 _e__q_). *2-(Piperazin-1-yl)ethyl:* 4.55–4.58 (4H, CH_piperazin_). *methoxypropyl:* 3.60–3.98 (NCH_2_CH_2_CH_2_OCH_3_).

^13^C NMR spectrum, ppm: *bicyclononan-9-one*: 46.9 (C-1,5), 58.5 (C-2,4,6,8), 214.8 (C-9). *2-(Piperazin-1-yl)ethyl*: 46.7 (CH_2piperazin_). *methoxypropyl:* 56.0, 27.8, 66.0, 71.4 (NCH_2_CH_2_CH_2_OCH_3_).

#### 4.1.3. Syntheses of β-Cyclodextrin Complexes of Bispidines (Compounds **4a**–**4e**)

*3-Boc-7-[3-(1H-imidazol-1-yl)propyl]*-*3,7-diazabicyclo[3.3.1]nonane* (**3a**). The procedure for the synthesis of 3-Boc-7-[3-(1H-imidazol-1-yl)propyl]-3,7-diazabicyclo[3.3.1]nonane (**3a**) was described earlier [35].

*3-(3-Hydroxypropyl)-7-[3-(1H-imidazol-1-yl)propyl]*-*3,7-diazabicyclo[3.3.1]nonane* (**3b**). A mixture of 2.0 g (0.006 mol) of 3-(3-hydroxypropyl)-7-[3-(*1H-*imidazol-1-yl)propyl]-3,7-diazabicyclo[3.3.1]nonan-9-one (***2b***) and 1 mL (0.031 mol) of hydrazine hydrate (99% solution) in 17.5 mL of triethylene glycol at 60 °C was added to 4.17 g (0.074 mol) of KOH. The reaction mixture was heated to 150 °C and stirred at this temperature for 5 h. At a temperature of 190–200 °C, water and excess hydrazine were removed by evaporation. After cooling, 29 mL of distilled water was added, extracted with diethyl ether, and dried over anhydrous MgSO_4_. The solvent was evaporated, and the obtained product was purified by column chromatography on Al_2_O_3_, benzene:dioxane 5:1. A total of 1.28 g (67%) of 3-(3-hydroxypropyl)-7-[3-(1*H-*imidazol-1-yl)propyl]-3,7-diazabicyclo[3.3.1]nonane (**3b**) was obtained as a pale yellow oil with R_f_ = 0.36 (Al_2_O_3_, benzene: isopropanol = 7:1).

Found, %: C 65.77, Н 9.61, N 19.20. C_16_H_28_N_4_O.

Calculated, %: C 65.75, Н 9.59, N 19.18.

IR spectrum, cm^−1^: 1121 (ν_C-__О__-__C_).

^1^H NMR spectrum, δ, ppm: *bicyclononane:* 2.03–2.11 (2 H, Н-1_eq_,5_eq_), 1.42–1.64 (2Н, H-9), 2.37 (4H, Н-2_ax_,4_ax_,6_ax_,8_ax_), 2.87–3.08 (4 H, Н-2_eq_,4_eq_,6_eq_,8_eq_). *1H-imidazol-1-yl)propyl*: 1.56 (2H, NCH_2_CH_2_CH_2_N), 3.05–3.08, 3.86 (4H, NCH_2_CH_2_CH_2_N), 7.45 (3H, CH_imidazole_), *hydroxypropyl:* 4.12 (1H, NCH_2_CH_2_CH_2_OH).

^13^C NMR spectrum, δ, ppm: *bicyclononane*: 30.2 (C-1,5), 36.3 (C-9), 58.4 (C-2,4,6,8). *(1H-imidazol-1-yl)propyl:* 32.8 (CH_2_CH_2_CH_2_), 51.6 (CH_2_CH_2_CH_2_), 45.6 (CH_2_CH_2_CH_2_), 118.9, 128.7, 137.2 (CH_imidazol_). *hydroxypropyl:* 27.8 (NCH_2_CH_2_CH_2_OH), 51.6, 59.8 (NCH_2_CH_2_CH_2_OH).

*3-(3-Methoxypropyl)-7-[3-(1H-imidazol-1-yl)propyl]*-*3,7-diazabicyclo[3.3.1]nonane* (**3c**). The procedure for the synthesis of 3-(3-methoxypropyl)-7-[3-(1H-imidazol-1-yl)propyl]-3,7-diazabicyclo[3.3.1]nonane (**3c**) was described earlier [34].

*3-(3-Methoxypropyl)-7-[2-(pyridin-2-yl)ethyl]-3,7-diazabicyclo[3.3.1]nonane* (**3d**). The procedure for the synthesis of 3-(3-methoxypropyl)-7-[2-(pyridin-2-yl)ethyl]-3,7-diazabicyclo[3.3.1]nonan-9-one (**3d**) was described earlier [36].

*3-(3-Methoxypropyl)-7-[2-(piperazin-1-yl)]-3,7-diazabicyclo[3.3.1]nonane* (**3e**). A mixture of 2.0 g (0.006 mol) of 3-(3-methoxypropyl)-7-[2-(piperazin-1-yl)ethyl]-3,7-diazabicyclo[3.3.1]nonan-9-one (**2e**) and 1 mL (0.031 mol) of hydrazine hydrate (99% solution) in 17.5 mL of triethylene glycol at 60 °C was added to 4.17 g (0.074 mol) of KOH. The reaction mixture was heated to 150 °C and stirred at this temperature for 5 h. At a temperature of 190–200 °C, water and excess hydrazine were removed by evaporation. After cooling, 29 mL of distilled water was added, extracted with diethyl ether, and dried over anhydrous MgSO_4_. The solvent was evaporated, and the obtained product was purified by column chromatography on Al_2_O_3_ and benzene:dioxane 5:1. A total of 1.21 g (63%) of 3-(3-methoxypropyl)-7-[2-(piperazin-1-yl)ethyl]-3,7-diazabicyclo[3.3.1]nonane (***3e***) was obtained as a pale yellow oil with R_f_ = 0.34 (Al_2_O_3_, benzene: isopropanol = 7:1).

Found, %: C 65.79, H 10.81, N 18.00. C_17_H_34_N_4_O.

Calculated, %: C 65.81, H 10.97, N 18.06.

IR spectrum, cm^−1^: 1075 (ν_C__-__О__-__C_), 3374 (ν_N-H_).

^1^H NMR spectrum, δ, ppm: *bicyclononan-9-one*: 1.59 (2H, Н-1_e__q_,5_e__q_), 2.42 (2H, Н-6_ax_,8_ax_), 2.98 (2H, Н-2_ax_,4_ax_), 2.94 (2 H, Н-6_e__q_,8 _e__q_), 3.04 (2H, Н-2 _e__q_,4 _e__q_). *2-(Piperazin-1-yl)ethyl:* 4.55–4.58 (4 H, CH_piperazin_). *methoxypropyl:* 1.13 (NCH_2_CH_2_CH_2_OCH_3_).

^13^C NMR spectrum, ppm: *bicyclononan-9-one*: 30.0 (C-1,5), 58.6 (C_2,4_), 58.4 (C_6,8_), 32,0 (C_9_). *2-(Piperazin-1-yl)ethyl:* 54,8, 53,4, 54,8, 46,3 (7N-CH_2_CH_2piperazine_). *methoxypropyl:* 56.3, 27.6, 66.0, 72.4 (NCH_2_CH_2_CH_2_OCH_3_).

*Complex3-Boc-7-[3-(1H-imidazol-1-yl)propyl]*-*3,7-diazabicyclo[3.3.1]nonane* (**4a**). The procedure for the synthesis of the complex of 3-Boc-7-[3-(1H-imidazol-1-yl)propyl]-3,7-diazabicyclo[3.3.1]nonane (**4a**) was described earlier [35].

*Complex of 3-(3-*hydroxypropyl*)-7*-[*3-(1H-imidazol-1-yl)propyl]-3,7-diazabicyclo[3.3.1]nonane* (**4b**). Hot solutions of 0.5 g (0.002 mol) of 3-(3-hydroxypropyl)-7-[3-(*1H-*imidazol-1-yl)propyl]-3,7-diazabicyclo[3.3.1]nonane (**3b**) in 25 mL of ethyl alcohol and 1.94 g (0.002 mol) of β-cyclodextrin in 30 mL of distilled water were mixed. The mixture was placed in a drying cupboard, and ethanol and water were evaporated at 50–55 °C to produce 2.25 g of compound **4b**.

Found, %: C 48.84, Н 6.84, N 3.95. C_58_H_98_N_4_O_36_.

Calculated, %: C 48.81, Н 6.87, N 3.93.

*Complex of 3-(3-methoxypropyl)-7-[3-(1H-imidazol-1-yl)propyl]*-*3,7-diazabicyclo[3.3.1]nonane* (**4c**). The procedure for the synthesis of the complex of 3-(3-methoxypropyl)-7-[3-(1H-imidazol-1-yl)propyl]-3,7-diazabicyclo[3.3.1]nonane (**4c**) was described earlier [34].

*Complex of 3-(3-methoxypropyl)-7-[2-(pyridin-2-yl)ethyl]-3,7-diazabicyclo[3.3.1]nonane* (**4d**). The procedure for the synthesis of the complex of 3-(3-methoxypropyl)-7-[2-(pyridin-2-yl)ethyl]-3,7-diazabicyclo[3.3.1]nonan-9-one (**4d**) was described earlier [36].

*Complex of 3-(3-methoxypropyl)-7-[2-(piperazin-1-yl)]-3,7-diazabicyclo[3.3.1]*nonane (**4e**). Hot solutions of 1.4 g (0.003 mol) of 3-(3-methoxypropyl)-7-[2-(piperazin-1-yl)]-3,7-diazabicyclo[3.3.1]nonane (**3e**) in 35 mL of ethyl alcohol and 4.0 g (0.003 mol) of β-cyclodextrin in 60 mL of distilled water were mixed. The mixture was placed in a drying cupboard, and ethanol and water were evaporated at 50–55 °C to produce 4.75 g of compound **4e**.

Found, %: C 49.01, H 7.14, N 3.91. C_59_H_104_N_4_O_36_.

Calculated, %: C 49.03, H 7.20, N 3.88.

#### 4.1.4. Syntheses of β-Cyclodextrin Complexes of o-Benzoyloximes of Bispidines (Compounds **7a**–**7e**)

*Oxime of 3-Boc-7-[3-(1H-imidazol-1-yl)propyl]-3,7-diazabicyclo[3.3.1]nonane-9-one* (**5a**). Five grams (0.014 mol) of 3-Boc-7-[3-(*1H-*imidazol-1-yl)propyl]-3,7-diazabicyclo[3.3.1]nonan-9-one (**2a**) in 85 mL of ethyl alcohol and 1.7 mL (0.021 mol) of pyridine were placed in a three-necked flask equipped with a mechanical stirrer, reflux condenser with a calcium chloride tube, and dropping funnel. While stirring, 2.53 g (0.036 mol) of hydroxylamine hydrochloride was added. The reaction mixture was heated at 110–120 °C for 27 h, the solvent was evaporated, and the residue was dissolved in 40 mL of water, alkalized with NaOH to pH 12, extracted with chloroform, and dried over anhydrous MgSO_4_. The solvent was evaporated, and the residue was purified by column chromatography on Al_2_O_3_, benzene:dioxane = 5:1. A total of 4.5 g (86%) of oxime of 3-Boc-7-[3-(1H-imidazol-1-yl)propyl]-3,7-diazabicyclo[3.3.1]nonan-9-one (**5a**) was obtained in the form of light yellow oils with R_f_ = 0.16 (Al_2_O_3_, benzene:isopropanol = 20:1).

Found, %: C 59.52; H, 8.00; N, 19.30. C_18_H_29_N_5_O_3_.

Calculated, %: C 59.50; H, 7.98; N, 19.28.

IR spectrum, cm^−1^: 1651 (ν_C=N_), 3380 (ν_О__-H_).

^1^Н NMR spectrum, δ, ppm: *bicyclononan-9-ketoxime*: 2.69 (2H, Н-1_eq_,5_eq_), 2.07–4.10 (4 H, Н-2_ax_,4_ax_,6_ax_,8_ax_), 2.60–4.72 (4H, Н-2_eq_,4_eq_,6_eq_,8_eq_), 6.96 (Н, N=OH). *1H-imidazol-1-yl)propyl*: 1.56 (2H, NCH_2_CH_2_CH_2_N), 3.13–3.22, 3.85 (4H, NCH_2_CH_2_CH_2_N), 6.96–7.49 (3H, CH_imidazol_). *Boc:* 1.48 (9H, CH_3_).

^13^C NMR ppm, δ, ppm: *bicyclononan-9-ketoxime*: 36.9 (C-5), 50.5 (C-6,8), 60.1 (C-2,4), 154.6 (C-9). *(1H-imidazol-1-yl)propyl*: 30.8 (CH_2_CH_2_CH_2_), 54.1 (CH_2_CH_2_CH_2_), 44.1 (CH_2_CH_2_CH_2_), 120.0 (CH_imidazol_), 128.7, 137.9. *Boc:* 28.6 (CH_3_), 79.1 (O-C), 158.3 (C=O).

*Oxime of 3-(3-*hydroxypropyl*)-7-[3-(1H-imidazol-1-yl)propyl]*-*3,7-diazabicyclo[3.3.1]nonane* (**5b**). A total of 6.0 g (0.020 mol) of 3-(3-hydroxypropyl)-7-[3-(*1H-*imidazol-1-yl)propyl]-3,7-diazabicyclo[3.3.1]nonan-9-one (**2a**) in 122 mL of ethyl alcohol and 2.37 g (0.030 mol) of pyridine were placed in a three-necked flask equipped with a mechanical stirrer, reflux condenser with a calcium chloride tube, and dropping funnel. While stirring, 3.61 g (0.052 mol) of hydroxylamine hydrochloride was added. The reaction mixture was heated at 110–120 °C for 29 h, the solvent was evaporated, and the residue was dissolved in 30 mL of water, alkalized with NaOH to pH 12, extracted with chloroform, and dried over anhydrous MgSO_4_. The solvent was evaporated, and the residue was purified by column chromatography on Al_2_O_3_, benzene:dioxane = 5:1. A total of 4.85 g (77%) of oxime of 3-(3-hydroxypropyl)-7-[3-(*1H-*imidazol-1-yl)propyl]-3,7-diazabicyclo[3.3.1]nonan-9-one (**5b**) was obtained in the form of light yellow oils with R_f_ = 0.07 (Al_2_O_3_, benzene:isopropanol = 20:1).

Found, %: C 59.79, H 8.40, N 21.84. C_16_H_27_N_5_O_2_.

Calculated, %: C 59.81, H 8.41, N 21.81.

IR spectrum, cm^−1^: 1510 (ν_C=N_), 3240 (ν_О__-H_).

^1^Н NMR spectrum, δ, ppm: *bicyclononan-9-ketoxime*: 1.60 (2 H, Н-1_eq_,5_eq_), 2.06–2.40 (4 H, Н-2_ax_,4_ax_,6_ax_,8_ax_), 2.99–3.42 (4 H, Н-2_eq_,4_eq_,6_eq_,8_eq_), 6.40 (Н, N=OH). *1H-imidazol-1-yl)propyl*: 1.60 (2H, NCH_2_CH_2_CH_2_N), 3.42, 3.85–3.87 (4H, NCH_2_CH_2_CH_2_N), 6.92, 7.23, 7.45 (3H, CH_imidazol_). *hydroxypropyl* 4.83 (1H, NCH_2_CH_2_CH_2_OH), 1.88–1.90 (2H, NCH_2_CH_2_CH_2_OH), 2.40, 3.68 (4H, NCH_2_CH_2_CH_2_OH).

^13^C NMR ppm, δ, ppm: *bicyclononan-9-ketoxime*: 42.2 (C-5), 57.7 (C-6,8), 58.1 (C-2,4), 160.9 (C-9). *(1H-imidazol-1-yl)propyl*: 27.7 (CH_2_CH_2_CH_2_), 50.0 (CH_2_CH_2_CH_2_), 45.4 (CH_2_CH_2_CH_2_), 119.0 (CH_imidazol_), 128.8, 137.1. *hydroxypropyl:* 27.7 (NCH_2_CH_2_CH_2_OH), 51.5, 59.5 (NCH_2_CH_2_CH_2_OH).

*Oxime of 3-(3-methoxypropyl)-7-[3-(imidazol-1-yl)propyl]-3,7-diazabicyclo[3.3.1]nonan-9-one* (**5c**)**.** A total of 3.5 g (0.008 mol) of 3-(3-methoxypropyl)-7-[3-(imidazol-1-yl)propyl]-3,7-diazabicyclo[3.3.1]nonan-9-one (***2c***) in 65 mL of ethyl alcohol and 1.3 mL (0.018 mol) of pyridine were placed in a three-necked flask equipped with a mechanical stirrer, reflux condenser with a calcium chloride tube, and dropping funnel. While stirring, 2.15 g (0.027 mol) of hydroxylamine hydrochloride was added. The reaction mixture was heated at 110–120 °C for 27 h, the solvent was evaporated, and the residue was dissolved in 27 mL of water, alkalized with NaOH to pH 12, extracted with chloroform, and dried over anhydrous MgSO_4_. The solvent was evaporated, and the residue was purified by column chromatography on Al_2_O_3_, benzene:dioxane = 5:1. 3.1 g (76%) of oxime of 3-(3-methoxypropyl)-7-[3-(imidazol-1-yl)propyl]-3,7-diazabicyclo[3.3.1]nonan-9-one (**5c**) was obtained in the form of light yellow oils with R_f_ = 0.21 (Al_2_O_3_, benzene:isopropanol = 20:1).

Found, %: C 60.86, H 9.72, N 20.95. C_17_H_33_N_5_O_2_.

Calculated, %: C 60.90, H 9.85, N 20.89.

IR spectrum, cm^−1^: 1659 (ν_C=N_), 3365 (ν_О-H_).

^1^Н NMR spectrum, δ, ppm: *bicyclononan-9-ketoxime*: 2.54 (2 H, Н-1_eq_,5_eq_), 2.12–3.78 (4 H, Н-2_ax_,4_ax_,6_ax_,8_ax_), 2.30–43.57 (4 H, Н-2_eq_,4_eq_,6_eq_,8_eq_), 6.96 (Н, N=OH). *1H-imidazol-1-yl)propyl*: 1.53 (2H, NCH_2_CH_2_CH_2_N), 3.16–3.20, 3.75 (4H, NCH_2_CH_2_CH_2_N), 6.96–7.49 (3H, CH_imidazol_). meth*oxypropyl:* 3.83 (3H, NCH_2_CH_2_CH_2_OCH_3_), 1.75 (2H, NCH_2_CH_2_CH_2_OCH_3_), 2.53, 3.74 (4H, NCH_2_CH_2_CH_2_OCH_3_).

^13^C NMR ppm, δ, ppm: *bicyclononan-9-ketoxime*: 37.9 (C-5), 53.5 (C-6,8), 65.1 (C-2,4), 161.5 (C-9). *(1H-imidazol-1-yl)propyl*: 28.8 (CH_2_CH_2_CH_2_), 45.9 (CH_2_CH_2_CH_2_), 44.8 (CH_2_CH_2_CH_2_), 115.4, 119.7, 127.5. (CH_imidazol_). *methoxypropyl:* 29.2 (CH_3_), 73.9 (O-C), 149.9 (C=O).

*O-Benzoyloxime of 3-Boc-7-[3-(1H-imidazol-1-yl)propyl]-3,7-diazabicyclo[3.3.1] nonan-9-one* (**6a**). Then, 4.42 g (0.012 mol) of oxime of 3-Boc-7-[3-(*1H-*imidazol-1-yl)propyl]-3,7-diazabicyclo[3.3.1]nonan-9-one (**5a**) was mixed with 84 mL of absolute benzene, and a mixture of 14 mL of absolute benzene and 2.8 mL (0.024 mol) of benzoyl chloride was added dropwise. The reaction took place at room temperature. Finally, 10 mL of distilled water was added to the reaction mixture and neutralized with potash to pH 10–11. The product was extracted with chloroform, and the combined extracts were dried over anhydrous MgSO_4_. The solvent was evaporated, and the residue was distilled in vacuo. A total of 2.66 g (50% of theory) of the O-benzoyloxime of 3-Boc-7-[3-(1H-imidazol-1-yl)propyl]-3,7-diazabicyclo[3.3.1]nonan-9-one (**6a**) was obtained in the form of a yellow oil (16), R_f_ = 0.56 (Al_2_O_3_, benzene:isopropanol = 7:1).

Found, %: C 64.27; H, 7.04; N, 15.01. C_25_H_33_N_5_O_4_.

Calculated, %: C 64.24; H, 7.07; N, 14.99.

IR spectrum, cm^−1^: 1725 (ν_C=__О_).

^1^Н NMR spectrum, δ, ppm: ester of bicyclononan-9-ketoxime: 2.77 (2 H, Н-1_eq_,5_eq_), 2.12–4.15 (4 H, Н-2_ax_,4_ax_,6_ax_,8_ax_), 2.65–4.77 (4 H, Н-2_eq_,4_eq_,6_eq_,8_eq_), 7.67–8.05 (5H, Ph). *1H-imidazol-1-yl)propyl*: 1.56 (2H, NCH_2_CH_2_CH_2_N), 3.13–3.22, 3.85 (4H, NCH_2_CH_2_CH_2_N), 6.96, 7.03, 7.49 (3H, CH_imidazol_). *Boc:* 1.48 (9H, CH_3_).

^13^C NMR spectrum, δ, ppm: ester of bicyclononan-9-ketoxime: 36.9, 39.9 (C-1,5), 45.5 60.0 (C-2,4,6,8), 164.7 (C-9). *(1H-imidazol-1-yl)propyl:* 29.4 (CH_2_CH_2_CH_2_), 50.1 (CH_2_CH_2_CH_2_), 47.9 (CH_2_CH_2_CH_2_), 120.5 (CH_imidazol_), 133.9, 136.3. *Boc:* 28.6 (CH_3_), 80.2 (O-C), 159.5 (C=O).

*O-Benzoyloxime of 3-(3-hydroxypropyl)-7-[3-(1H-imidazol-1-yl)propyl]-3,7-diazabicyclo[3.3.1]nonan-9-one* (**6b**). Oxime (0.014 mol) of 3-(3-hydroxypropyl)-7-[3-(*1H-*imidazol-1-yl)propyl]-3,7-diazabicyclo[3.3.1]nonan-9-one (**5b**) was mixed with 100 mL of absolute benzene, and a mixture of 14 mL of absolute benzene and 3.3 mL (0.014 mol) of benzoyl chloride was added dropwise. The reaction took place at room temperature. Finally, 10 mL of distilled water was added to the reaction mixture and neutralized with potash to pH 10–11. The product was extracted with chloroform, and the combined extracts were dried over anhydrous MgSO_4_. The solvent was evaporated, and the residue was distilled in vacuo. A total of 4.01 g (70% of theory) of O-benzoyloxime of 3-(3-hydroxypropyl)-7-[3-(*1H-*imidazol-1-yl)propyl]-3,7-diazabicyclo[3.3.1]nonan-9-one (**6b**) was obtained in the form of a yellow oil, R_f_ = 0.64 (Al_2_O_3_, benzene:isopropanol = 7:1).

Found, %: C 64.90, H 7.32, N 16.45. C_23_H_31_N_5_O_3_.

Calculated, %: C 64.94, H 7.29, N 16.47.

IR spectrum, cm^−1^: 1737 (ν_C=__О_).

^1^Н NMR spectrum, δ, ppm: ester of bicyclononan-9-ketoxime: 2.74 (2H, Н-1_eq_,5_eq_), 2.11–2.24 (4 H, Н-2_ax_,4_ax_,6_ax_,8_ax_), 2.65–2.77 (4H, Н-2_eq_,4_eq_,6_eq_,8_eq_), 7.67–8.05 (5H, Ph). *1H-imidazol-1-yl)propyl*: 1.56 (2H, NCH_2_CH_2_CH_2_N), 3.13–3.22, 3.85 (4H, NCH_2_CH_2_CH_2_N), 6.96, 7.03, 7.49 (3H, CH_imidazol_). *hydroxypropyl:* 4.83 (1H, NCH_2_CH_2_CH_2_OH), 1.75 (2H, NCH_2_CH_2_CH_2_OH), 2.56, 3.76 (4H, NCH_2_CH_2_CH_2_OH).

^13^C NMR spectrum, δ, ppm: ester of bicyclononan-9-ketoxime: 37.7–40.7 (C-1,5), 59.1–59.4 (C-2,4,6,8), 167.6 (C-9). *(1H-imidazol-1-yl)propyl:* 29.4 (CH_2_CH_2_CH_2_), 50.1 (CH_2_CH_2_CH_2_), 48.0 (CH_2_CH_2_CH_2_), 120.5 (CH_imidazol_), 133.9, 136.3. *hydroxypropyl:* 28.1 (NCH_2_CH_2_CH_2_OH), 52.4, 60.6 (NCH_2_CH_2_CH_2_OH).

*O-benzoyloxime of 3-(3-methoxypropyl)-7-[3-(imidazol-1-yl)propyl]-3,7-diazabicyclo[3.3.1]nonan-9-one* (**6c**). Three grams (0.009 mol) of oxime of 3-(3-methoxypropyl)-7-[3-(imidazol-1-yl)propyl]-3,7-diazabicyclo[3.3.1]nonan-9-one (**5c**) was mixed with 90 mL of absolute benzene, and a mixture of 12 mL of absolute benzene and 2 mL (0.014 mol) of benzoyl chloride was added dropwise. The reaction took place at room temperature. Finally, 8 mL of distilled water was added to the reaction mixture and neutralized with potash to pH 10–11. The product was extracted with chloroform, and the combined extracts were dried over anhydrous MgSO_4_. The solvent was evaporated, and the residue was distilled in vacuo. A total of 3.05 g (64% of theory) of O-benzoyloxime of 3-(3-methoxypropyl)-7-[3-(imidazol-1-yl)propyl]-3,7-diazabicyclo[3.3.1]nonan-9-one (**6c**) was obtained in the form of a yellow oil, R_f_ = 0.61 (Al_2_O_3_, benzene:isopropanol = 7:1).

Found, %: C 60.86, H 9.72, N 20.95. C_17_H_33_N_5_O_2_.

Calculated, %: C 60.90, H 9.85, N 20.89.

IR spectrum, cm^−1^: 1734 (ν_C=__О_).

^1^Н NMR spectrum, δ, ppm: ester of bicyclononan-9-ketoxime: 2.71 (2 H, Н-1_eq_,5_eq_), 2.01–2.14 (4 H, Н-2_ax_,4_ax_,6_ax_,8_ax_), 2.35–2.45 (4 H, Н-2_eq_,4_eq_,6_eq_,8_eq_), 7.67–8.05 (5H, Ph). *1H-imidazol-1-yl)propyl*: 1.56 (2H, NCH_2_CH_2_CH_2_N), 3.13–3.22, 3.85 (4H, NCH_2_CH_2_CH_2_N), 6.96, 7.03, 7.49 (3H, CH_imidazol_). *hydroxypropyl:* 4.83 (1H, NCH_2_CH_2_CH_2_OH), 1.75 (2H, NCH_2_CH_2_CH_2_OH), 2.56, 3.76 (4H, NCH_2_CH_2_CH_2_OH).

^13^C NMR ppm, δ, ppm: *bicyclononan-9-ketoxime*: 36.0 (C-5), 51.9 (C-6,8), 64.5 (C-2,4), 159.5 (C-9). *(1H-imidazol-1-yl)propyl*: 27.5 (CH_2_CH_2_CH_2_), 43.9 (CH_2_CH_2_CH_2_), 45.2 (CH_2_CH_2_CH_2_), 116.2, 118.4, 129.2. (CH_imidazol_). *methoxypropyl:* 27.4 (CH_3_), 75.1 (O-C).

*Complex of O-benzoyloxime of 3-Boc-7-[3-(1H-imidazol-1-yl)propyl]-3,7-diazabicyclo[3.3.1]nonan-9-one* (**7a**). Hot solutions of 1.0 g (0.002 mol) of O-benzoyloxime of 3-Boc-7-[3-(*1H-*imidazol-1-yl)propyl]-3,7-diazabicyclo[3.3.1]nonan-9-one (**6a**) in 25 mL of ethyl alcohol and 3.124 g (0.002 mol) of β-cyclodextrin in 40 mL of distilled water were mixed together. The mixture was placed in a drying cupboard, and ethanol and water were evaporated at 50–55 °C to produce 3.57 g of compound **7a**.

Found, %: C 50.25, H 6.46, N 4.35. C_67_H_103_N_5_O_39_.

Calculated, %: C 50.22, H 6.43, N 4.37.

*Complex of O-benzoyloxime of3-(3-hydroxypropyl)-7-[3-(1H-imidazol-1-yl)propyl]-3,7-diazabicyclo[3.3.1]nonan-9-one* (**7b**). Hot solutions of 1.5 g (0.003 mol) of O-benzoyloxime of 3-(3-hydroxypropyl)-7-[3-(*1H-*imidazol-1-yl)propyl]-3,7-diazabicyclo[3.3.1]nonan-9-one (**6b**) in 35 mL of ethyl alcohol and 4 g (0.003 mol) of β-cyclodextrin in 60 mL of distilled water were mixed together. The mixture was placed in a drying cupboard, and ethanol and water were evaporated at 50–55 °C to produce 4.98 g of compound **7b**.

Found, %: C 50.01, H 6.45, N 4.51. C_65_H_101_N_5_O_38_.

Calculated, %: C 50.03, H 6.48, N 4.49.

*Complex of O-benzoyloxime of3-(3-methoxypropyl)-7-[3-(1H-imidazol-1-yl)propyl]-3,7-diazabicyclo[3.3.1]nonan-9-one* (**7c**). Hot solutions of 2.5 g (0.006 mol) of O-benzoyloxime of 3-(3-methoxypropyl)-7-[3-(1H-imidazol-1-yl)propyl]-3,7-diazabicyclo[3.3.1]nonan-9-one (**6c**) in 45 mL of ethyl alcohol and 6.8 g (0.006 mol) of β-cyclodextrin in 100 mL of distilled water were mixed together. The mixture was placed in a drying cupboard, and ethanol and water were evaporated at 50–55 °C to produce 8.8 g of compound **7c**.

Found, %: C 49.81, H 6.52, N 4.31. C_66_H_103_N_5_O_39_.

Calculated, %: C 49.84, H 6.48, N 4.40.

### 4.2. Biological Experimental Part

#### 4.2.1. The Preparation of Regenerating Liver Homogenates

The regenerating rat liver was used as the source of the enzymes of PA catabolism [33]. The study was approved by the local Ethics Committee of RUDN University (protocol no. 17/09-2015). Six-month-old female Sprague–Dawley rats (RUDN vivarium) were subjected to partial hepatectomy according to standard surgical techniques [50]. Eighteen hours after the operation, regenerating livers were homogenized with an Omni MultiMix200 homogenizer (Omni, Kennesaw, GA, USA) in two volumes of ice-cold 50 mM Tris-HCl buffer, pH = 9.0, containing 0.05% SDS (Sigma-Aldrich, St. Louis, MO, USA) and protease inhibitors Complete™ Protease Inhibitor Cocktail (Roche, Basel, Switzerland). The homogenates were centrifuged at 10,000× *g* for 20 min at +4 °C, and the supernatant was used as a source of polyamine oxidases in their natural environment for the following in vitro evaluation of the action of bispidines on their activities.

#### 4.2.2. Determination of Amine Oxidase Activity in Rat Liver Homogenates

Determination of amine oxidase activity in the presence of the tested compounds was performed in rat liver homogenates, as described previously [33]. A 30% liver homogenate in 50 mM Tris-HCl buffer, pH = 9.0, was incubated in 96-well plates for 1 h at 37 °C with 150 U/mg peroxidase (Sigma-Aldrich, St. Louis, MO, USA) in the presence of 10 μM test compounds. O-dianisidine (Sigma-Aldrich), Spd·3HCI (Sigma-Aldrich, St. Louis, MO, USA) and Spm·4HCI (Sigma-Aldrich, St. Louis, MO, USA) were then added to a final concentration of 10.7 μM. The mixture was incubated again for 30 min, and the optical density was measured at 540 nm (Novaspec III, Amersham Biosciences, Amersham, UK). The activity of polyamine oxidases in samples was assessed based on the calibration curve per unit of protein. The protein assay was performed according to the Bradford method [51].

#### 4.2.3. Cell Culture

Humаn hepаtocellulаr cаrcinomа HepG2 cell line (АTCC, Mаnаssаs, VА, USA) wаs grown in RPMI-1640 medium (Gibco, Thermo Fisher Scientific Inc., Wаlthаm, MА, USA). Normаl humаn fibroblаsts WI-38 (АTCC, Mаnаssаs, VА, USA) were grown in DMЕM medium (Gibco, Thermo Fisher Scientific Inc., Wаlthаm, MА, USA) and were used as control noncancer cells. Both cell lines were passage number 4. Аll mediа were supplemented with 5% fetаl bovine serum (Cаpricorn Scientific, Еbsdorfergrund, Germаny) аnd 1% of sodium pyruvаte (Pаneco, Moscow, Russiа). Cells were grown аt 5% CО2/95% аir in а humidified аtmosphere аt 37 °C to confluency 80–90%. To harvest, the cells were washed up to three times with 5 mL prewarmed phosphate-buffered saline (Paneco, Moscow, Russia) and detached with prewarmed 0.25% trypsin/EDTA (Gibco, Thermo Fisher Scientific Inc., Waltham, MA, USA). Cell lines were tested for mycoplаsmа contаminаtion before the experiment using Mycoplаsmа Detection Kit PlаsmoTest™ (InvivoGen, Sаn Diego, CА, USA).

#### 4.2.4. Cell Viability Testing

To determine the influence of the compounds on cell viability, an MTT assay was performed [52]. Briefly, cells were removed from culture flasks by trypsinization and seeded in 96-well plаtes (TPP, Trаsаdingen, Switzerlаnd) at a concentration of 1 × 10^4^ cells per well. In 24 h, synthesized compounds within the rаnge of concentrаtions 1–50 µM were added and incubated for 72 h. Tetrаzolium sаlt (3-(4,5-dimethyl-thiаzol-2-yl)-2,5-diphenyltetrаzolium bromide, Servа, Heidelberg, Germаny) solution (10 µL) was added to each well to reach a final concentration of 0.45 mg/mL and incubated at 37 °C for 4 hr. Аfter incubаtion, the formаzаn crystаls were dissolved in 100 µL dimethyl sulfoxide (DMSO), аnd the аbsorbаnce wаs meаsured аt 570 nm with а CLARIOstar Plus multiplаte reаder (BMG Labtech, Ortenberg, Germany). The percentage of cell viability was calculated relative to nontreated control cells.

The mаximum nontoxic concentrаtion (MNTC) wаs considered the highest concentrаtion of the compound thаt produced no stаtisticаlly significаnt increаse in cell viability. Compound concentrаtions thаt produce 50% of cell viаbility (IC50) were cаlculаted from curves constructed by plotting cell viаbility (%) versus drug concentrаtion (µM) [53]. To test the elevаtion of toxicity in the presence of PАs, cells were incubаted with MNTC of 4c or 4e in the presence of 1 or 10 µM Spm·4HCI or Spd·3HCI (both from Sigmа–Аldrich, St. Louis, MО, USA).

#### 4.2.5. Detection of Аpoptosis

To evаluаte аpoptosis, the incubаted cells were re-suspended in phosphаte sаline buffer (Pаneco, Moscow, Russiа) аnd incubаted with Аnnexin V-FITC аnd propidium iodide (PI) from а FITC Аnnexin V/Deаd Cell Аpoptosis kit (Life Technologies, Cаrlsbаd, CА, USA), аccording to the mаnufаcturer’s protocol. The counting of 5 × 10^4^ cells аt eаch point wаs performed by flow cytometry with а MАCS Quаnt Аnаlyzer 10 (Miltenyi Biotec, Bergisch Glаdbаch, Germаny) аs it wаs previously described [54].

#### 4.2.6. Stаtistics

SPSS 25 softwаre (IBM SPSS Stаtistics, Аrmonk, NY) wаs used for stаtisticаl аnаlysis. The dаtа on PА cаtаbolism influence belonged to the Gаussiаn distribution model, and pаrаmetricаl methods were аpplied. The results аre presented аs meаn ± stаndаrd error of meаn (SЕM). The dаtа on cytotoxicity аnd аpoptosis evаluаtion did not belong to the Gаussiаn distribution model; non-pаrаmetricаl methods were аpplied. Compаrisons between compounds were performed using Mаnn–Whitney U test with correction for multiple testing аccording to Bonferroni method. Differences described by *p* ≤ 0.05 were considered significаnt. The results аre presented аs meаn ± stаndаrd error of meаn (SЕM).

## 5. Conclusions

The enhancement of PA catabolism in cancer cells has become a promising approach for the development of antitumor therapy. Synthetized bispidine derivative 4e 3-(3-methoxypropyl)-7-[3-(1H-piperazin-1-yl)ethyl]-3,7-diazabicyclo[3.3.1]nonane demonstrated the ability to activate PA catabolism in regenerating rat liver homogenates. This compound could significantly decrease the viability of cancer cells in vitro. Adding Spd or Spm as a substrate of oxidation to boost toxicity, provided convincing evidence for the anticancer potential of bispidines. Minimally toxic bispidines strongly synergized the potency of the two PAs in the tumor cell line but not in normal fibroblasts. We can conclude that the lead compound 4e can become a potential anticancer drug substance which mechanism relies on the induction of PA catabolism in tumor cells.

## Figures and Tables

**Table 1:** The determined IC50 values (µM) for cancer and normal cell lines.

Cell Line	Compound								
	4a	4b	4c	4d	4e	7a	7b	7c	Cisplatin
HepG2	16.0	12.7	9.3	12.5	3.5	15.6	11.1	6.6	15.9 *
WI-38	13.1	10.1	13.8	4.6	5.1	24.3	5.0	6.6	18.5 **

The results are presented as the mean from eight different MTT tests. (*N* = 8). Errors were in the range of ±5% of the reported values. Literature data: * [38], ** [39].

**Table 2:** Literature data on PA metabolism in the experimental cell lines and in cell lines of theoretical comparison.

	PA Metabolism Marker	Experimental Cell Lines		Cell Lines of Theoretical Comparison	
		WI-38	HepG2	Normal Hepatocytes (Epithelial)	Fibroblasts (Mesenchymal Cells)
PA levels	Spd, nmol/mg of protein	7 *	5.1 **	NF	NF
	Spm, nmol/mg of protein	9.1 *	8.2 **	NF	NF
PA Synthesis	ODC transcription level, nTPM	NF	429.9 #	50.3 #	63.2 #
	S-AdoMetDC transcription level, nTPM	NF	96.5 #	59.3 #	132.1 #
PA Degradation	SSAT transcription level, nTPM	NF	503.5 #	994.1 #	1211.3 #
	APAO transcription level, nTPM	NF	0.0 #	13.4 #	3.6 #
	SMOX transcription level, nTPM	NF	50.8 #	2.3 #	11.3 #

# www.proteinatlas.org (accessed on 12 November 2021), NF—not found, * literature data: [48] ** literature data: [49].

## Data Availability

The datasets used and/or analyzed during the present study are available from the corresponding author on reasonable request.

## Supplementary Materials

The following supporting information can be downloaded at https://www.mdpi.com/article/10.3390/molecules27123872/s1; Figure S1. IR spectrum of 1-(3-hydroxypropyl)piperidin-4-one (1 b).; Figure S2. IR spectrum of 3-(3-hydroxypropyl)-7-[3-(1H-imidazol-1-yl)propyl]-3,7-diazabicyclo[3.3.1]nonan-9-one (2b).; Figure S3. IR spectrum of 3-(3-hydroxypropyl)-7-[3-(1H-imidazol-1-yl)propyl]-3,7-diazabicyclo[3.3.1]nonane (3b).; Figure S4. IR spectrum of oxime 3-Boc-7-[3-(1H-imidazol-1-yl)propyl]-3,7-diazabicyclo[3.3.1]nonan-9-one (5a).; Figure S5. IR spectrum of oxime of 3-(3-hydroxypropyl)-7-[3-(1H-imidazol-1-yl)propyl]-3,7-diazabicyclo[3.3.1]nonan-9-one (5b).; Figure S6. IR spectrum of O-benzoyloxime 3-(3-Boc)-7-[3-(1H-imidazol-1-yl)propyl]-3,7-diazabicyclo[3.3.1]nonan-9-one (6a).; Figure S7. IR spectrum of O-benzoyl oxime of 3-(3-hydroxypropyl)-7-[3-(1H-imidazol-1-yl)propyl]-3,7-diazabicyclo[3.3.1]nonan-9-one (6b).; Figure S8. 1H NMR spectrum of 1-(3-hydroxypropyl)piperidin-4-one (1b)_in_CHCl_3_.; Figure S9. 13C NMR spectrum of 1-(3-hydroxypropyl)piperidin-4-one (1b)_in_CHCl_3_.; Figure S10. 1H NMR spectrum of 3-(3-hydroxypropyl)-7-[3-(1H-imidazol-1-yl)propyl]-3,7-diazabicyclo[3.3.1]nonan-9-one (2b)_in_CHCl_3_.; Figure S11. 13C NMR spectrum of 3-(3-hydroxypropyl)-7-[3-(1H-imidazol-1-yl)propyl]-3,7-diazabicyclo[3.3.1]nonan-9-one (2b)_in_CHCl3.; Figure S12. 1H NMR spectrum of 3-(3-hydroxypropyl)-7-[3-(1H-imidazol-1-yl)propyl]-3,7-diazabicyclo[3.3.1]nonane(3b)_in_CHCl3.; Figure S13. 13C NMR spectrum of 3-(3-hydroxypropyl)-7-[3-(1H-imidazol-1-yl)propyl]-3,7-diazabicyclo[3.3.1]nonane(3b)_in_CHCl3.; Figure S14. 1H NMR spectrum of oxime of 3-(3-Boc)-7-[3-(1H-imidazol-1-yl)propyl]-3,7-diazabicyclo[3.3.1]nonan-9-one (5a)_in_DMSO.; Figure S15. 13C NMR spectrum of oxime of 3-(3-Boc)-7-[3-(1H-imidazol-1-yl)propyl]-3,7-diazabicyclo[3.3.1]nonan-9-one (5a)_in_DMSO.; Figure S16. 1H NMR spectrum of oxime of 3-(3-hydroxypropyl)-7-[3-(1H-imidazol-1-yl)propyl]-3,7-diazabicyclo[3.3.1]nonan-9-one (5b)_in_CHCl3.; Figure S17. 13C NMR spectrum of oxime of 3-(3-hydroxypropyl)-7-[3-(1H-imidazol-1-yl)propyl]-3,7-diazabicyclo[3.3.1]nonan-9-one (5b)_in_CHCl3.; Figure S18. 1H NMR spectrum of O-benzoyloxime of 3-(3-Boc)-7-[3-(1H-imidazol-1-yl)propyl]-3,7-diazabicyclo[3.3.1]nonan-9-one (6a)_in_DMSO.; Figure S19. 13C NMR spectrum O-benzoyloxime of 3-(3-Boc)-7-[3-(1H-imidazol-1-yl)propyl]-3,7-diazabicyclo[3.3.1]nonan-9-one (6a)_in_DMSO.; Figure S20. 1H NMR spectrum of O-benzoyloxime of 3-(3-hydroxypropyl)-7-[3-(1H-imidazol-1-yl)propyl]-3,7-diazabicyclo[3.3.1]nonan-9-one (6b)_in_CHCl3.; Figure S21. 13C NMR spectrum of O-benzoyloxime of 3-(3-hydroxypropyl)-7-[3-(1H-imidazol-1-yl)propyl]-3,7-diazabicyclo[3.3.1]nonan-9-one (6b)_in_CHCl3.

## Sample Availability

Samples of the compounds are available from the authors upon reasonable request.
