# Supplementary material for: Anticancer Cytotoxic Activity of Bispidine Derivatives Associated with the Increasing Catabolism of Polyamines
Source: Molecules. 2022 Jun 16;27(12):3872. doi: 10.3390/molecules27123872 (PMC9229528; doi:10.3390/molecules27123872)
Supplement: Supplementary file 1 [file molecules-27-03872-s001.zip › molecules-1772261-supplementary.pdf]

Article

Anticancer Cytotoxic Activity of Bispidine Derivatives Associated with the Increasing Catabolism of Polyamines

Ekaterina V. Neborak [1,*], Altynay B. Kaldybayeva [2,3], Lylia Bey [1], Aigul Y. Malmakova [2], Anna S. Tveritinova [1], Abdullah Hilal [1,4], Valentina K. Yu [2], Maria V. Ploskonos [5], Marina V. Komarova [6], Enzo Agostinelli [7,8] and Dmitry D. Zhdanov [1,4,*]

1 Department of Biochemistry, Peoples' Friendship University of Russia (RUDN University), 8 Miklukho-Maklaya St., Moscow, 117198, Russian Federation, neborak_ev@pfur.ru, katevladis@mail.ru
2 A.B. Bekturov Institute of Chemical Sciences, Laboratory of Chemistry of Synthetic and Natural Medicinal Substances, Almaty, Kazakhstan, malmakova@mail.ru, altin_28.94@mail.ru, yu_vk@mail.ru
3 Al-Farabi Kazakh National University, Almaty, Kazakhstan, altin_28.94@mail.ru
4 Institute of Biomedical Chemistry, 10/8 Pogodinskaya St., 119121, Moscow, Russia, zhdanovdd@mail.ru
5 Astrakhan State Medical University of The Healthcare Ministry of Russian Federation, Astrakhan 414000, Russian Federation, ploskonoz@mail.ru
6 Samara University, Moskovskoye Sh., 34, 443086, Samara, Russian Federation, marinakom@yandex.ru
7 Department of Sense Organs, Faculty of Medicine and Dentistry, SAPIENZA University of Rome, University Hospital Policlinico Umberto I, I-00161 Rome, Italy, enzo.agostinelli@uniroma1.it
8 International Polyamines Foundation, ETS-ONLUS, I-00159 Rome, Italy, enzo.agostinelli@uniroma1.it

* Correspondence: neborak_ev@pfur.ru, katevladis@mail.ru; zhdanovdd@mail.ru

Supplementary Material

Spectral NMR data section ……………………………….Figures S2–S8
Spectral IR data section……………………………………Figures S9–S21

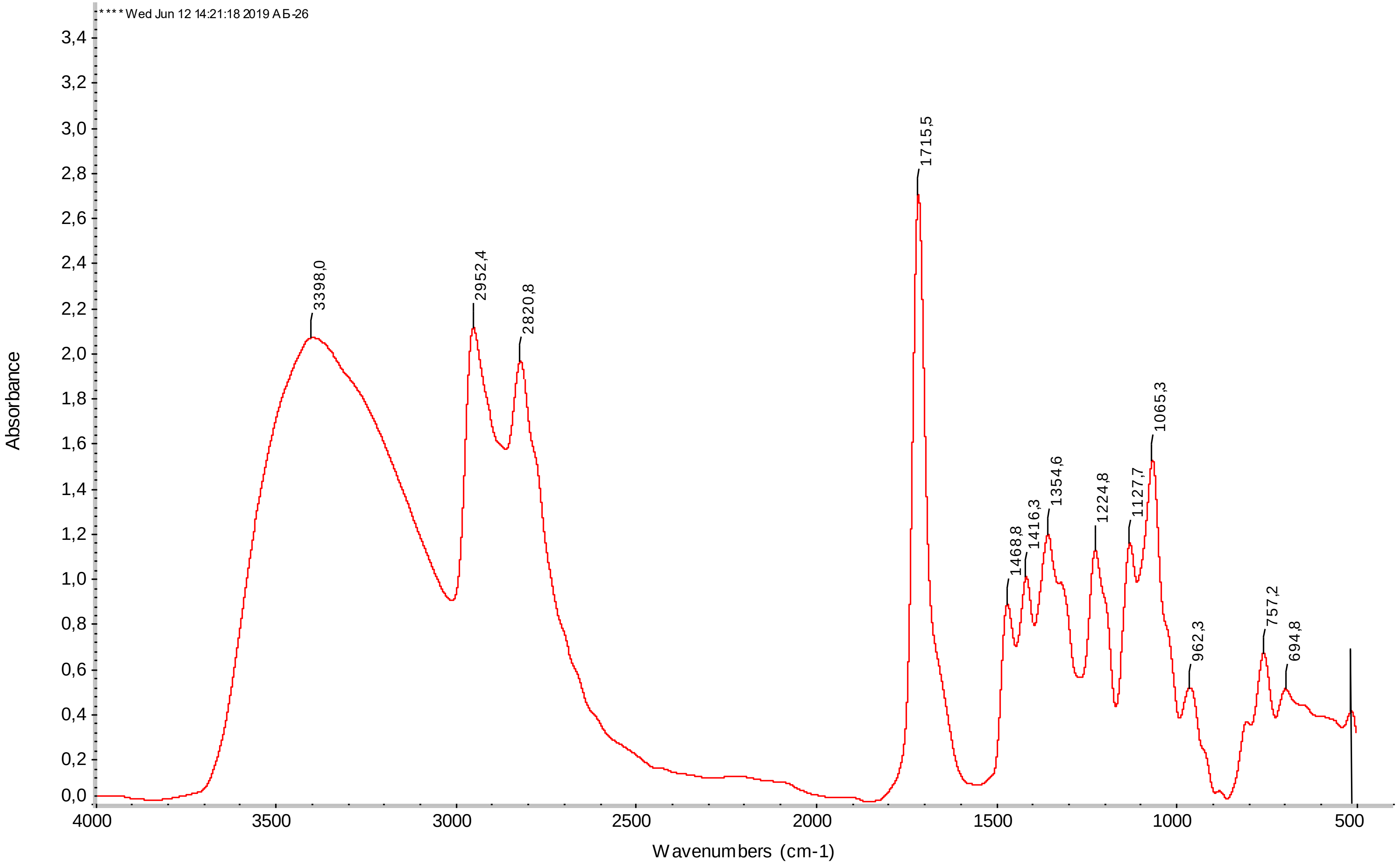

Figure S1. IR spectrum of 1-(3-hydroxypropyl)piperidin-4-one (1 b)

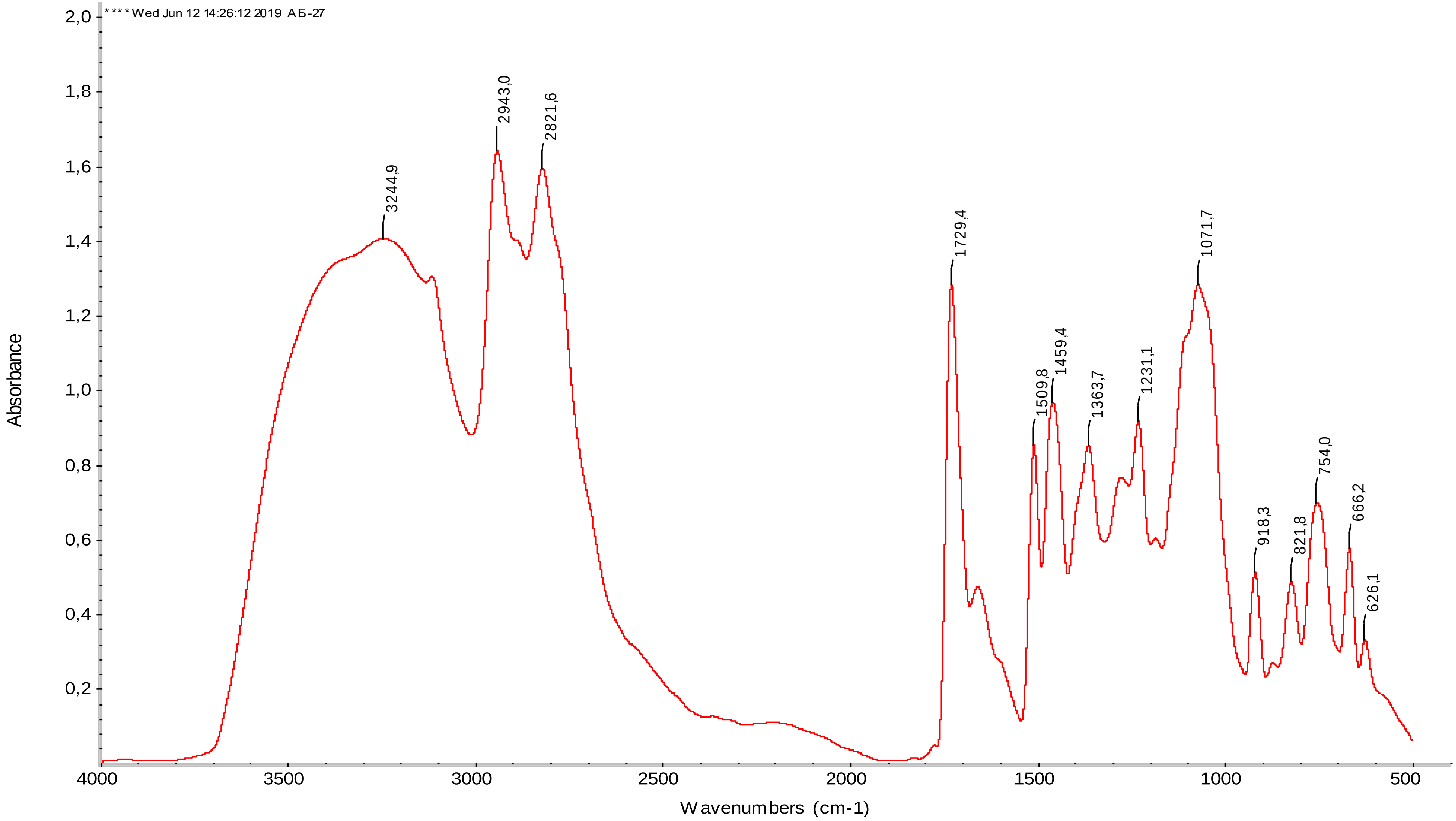

Figure S2. IR spectrum of 3-(3-hydroxypropyl)-7-[3-(1*H*-imidazol-1-yl)propyl]-3,7-diazabicyclo[3.3.1]nonan-9-one (2b)

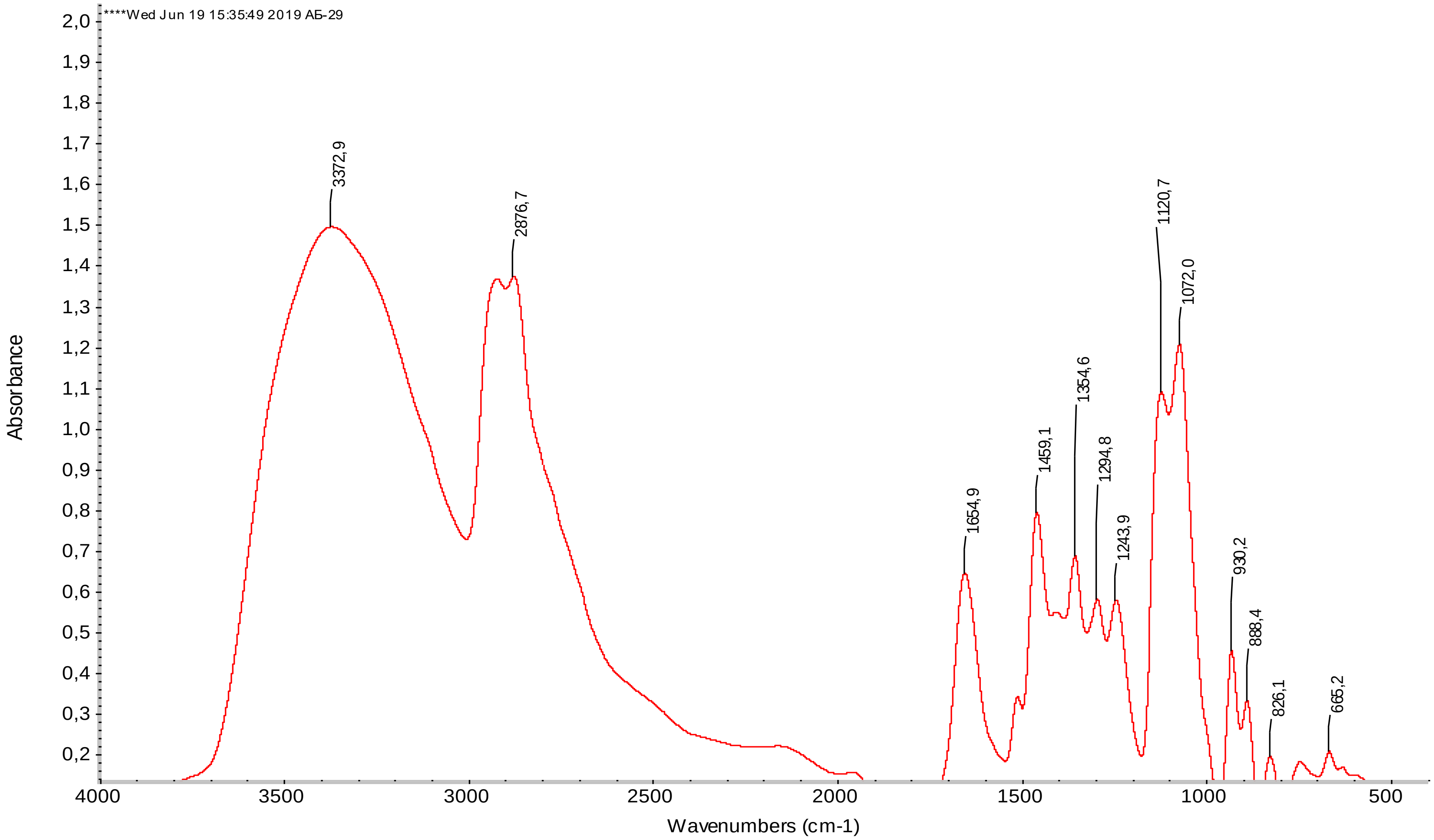

Figure S3. IR spectrum of 3-(3-hydroxypropyl)-7-[3-(1*H*-imidazol-1-yl)propyl]-3,7-diazabicyclo[3.3.1]nonane (3b)

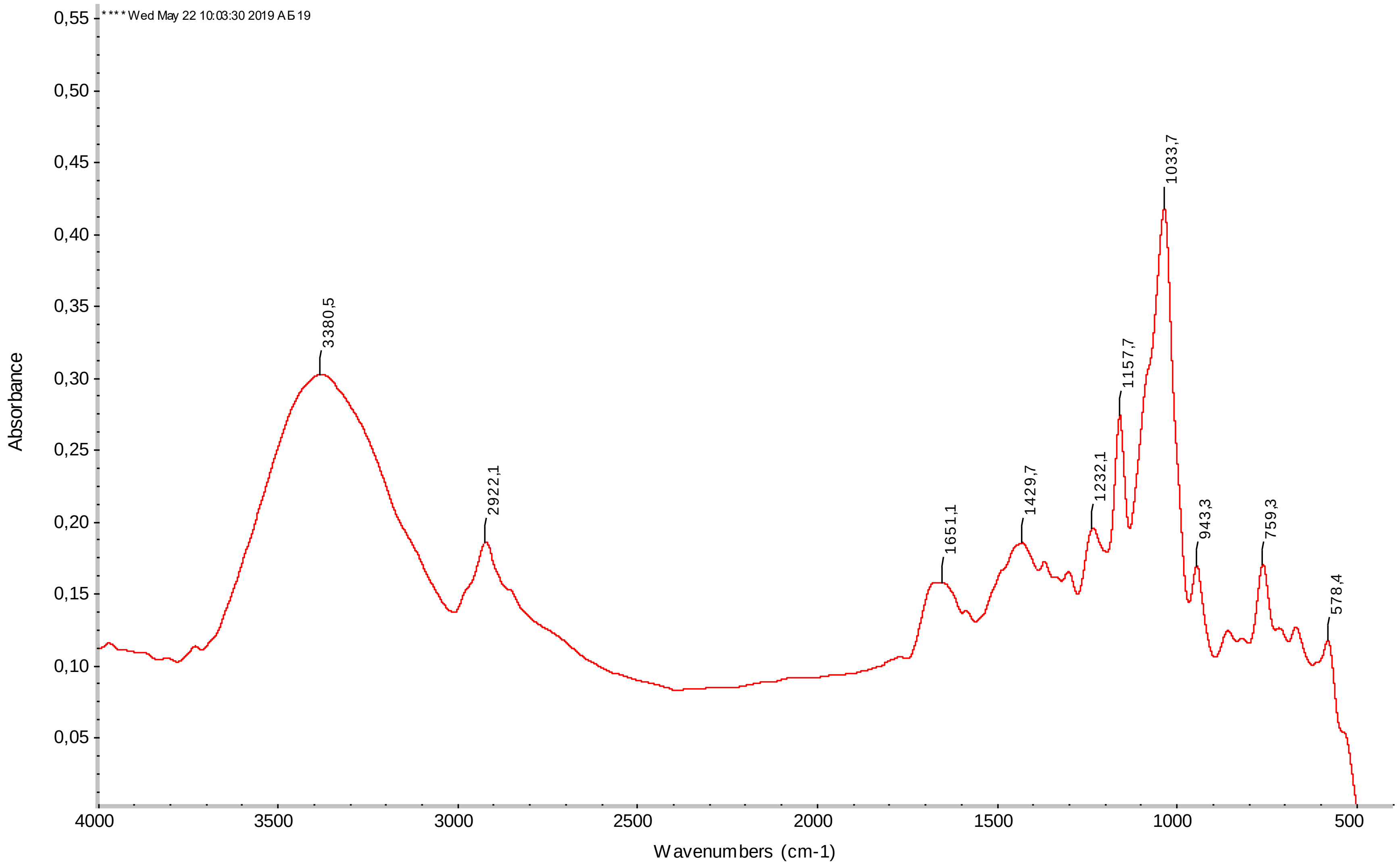

Figure S4. IR spectrum of oxime 3-Boc-7-[3-(1*H*-imidazol-1-yl)propyl]-3,7-diazabicyclo[3.3.1]nonan-9-one (5a)

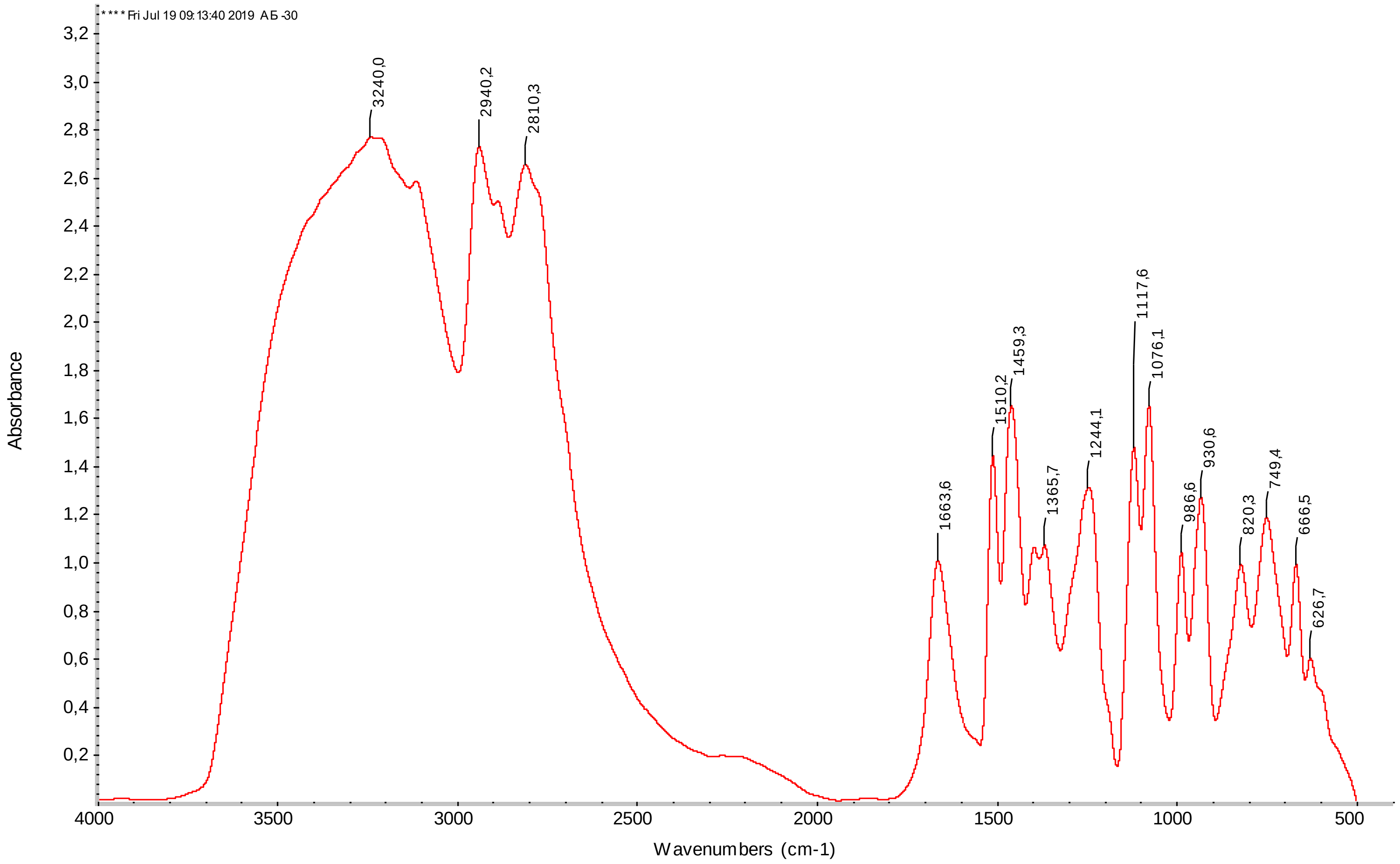

Figure S5. IR spectrum of oxime of 3-(3-hydroxypropyl)-7-[3-(*1H*-imidazol-1-yl)propyl]-3,7-diazabicyclo[3.3.1]nonan-9-one (5b)

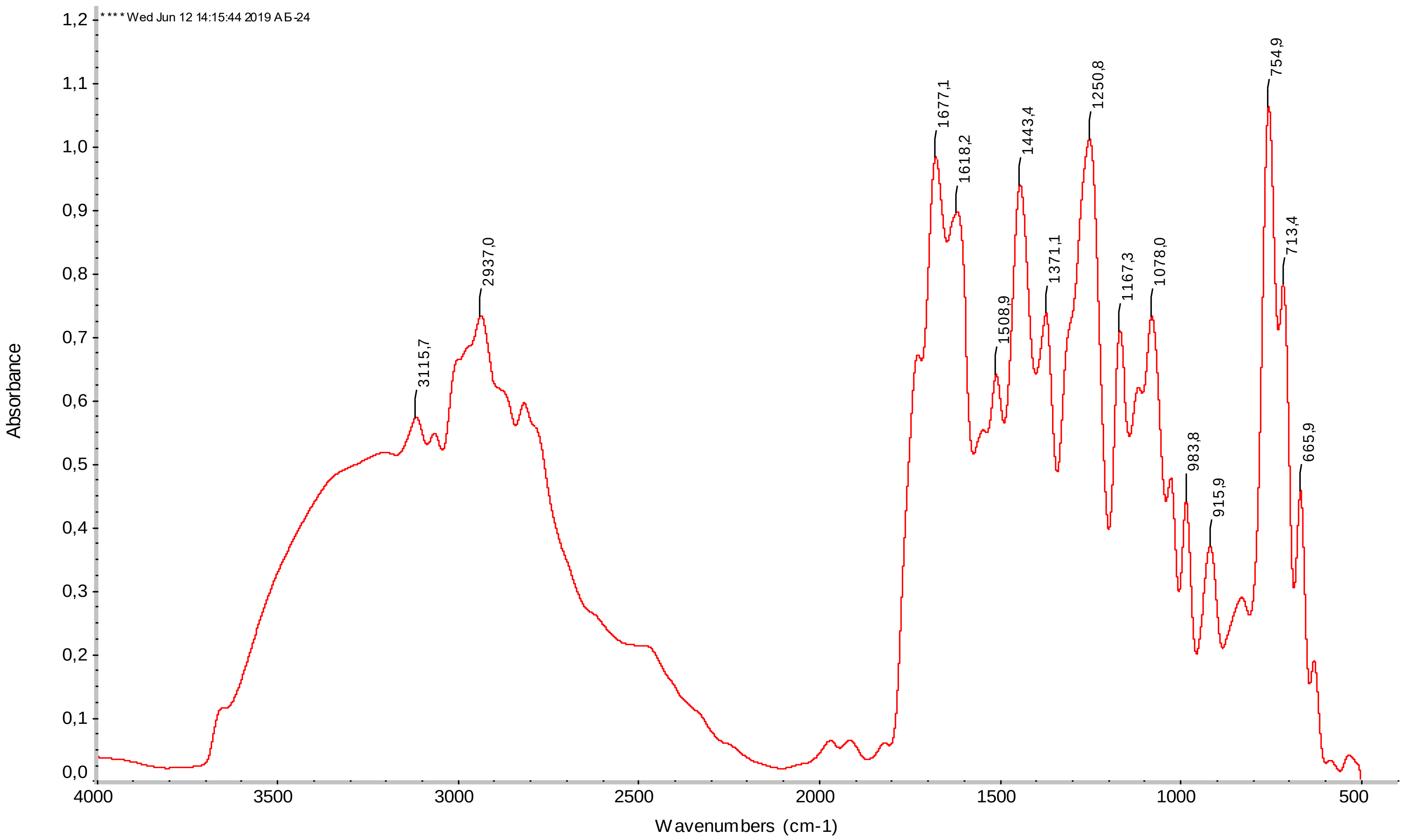

Figure S6. IR spectrum of O-benzoyloxime 3-(3-Boc)-7-[3-(1*H*-imidazol-1-yl)propyl]-3,7-diazabicyclo[3.3.1]nonan-9-one (6a)

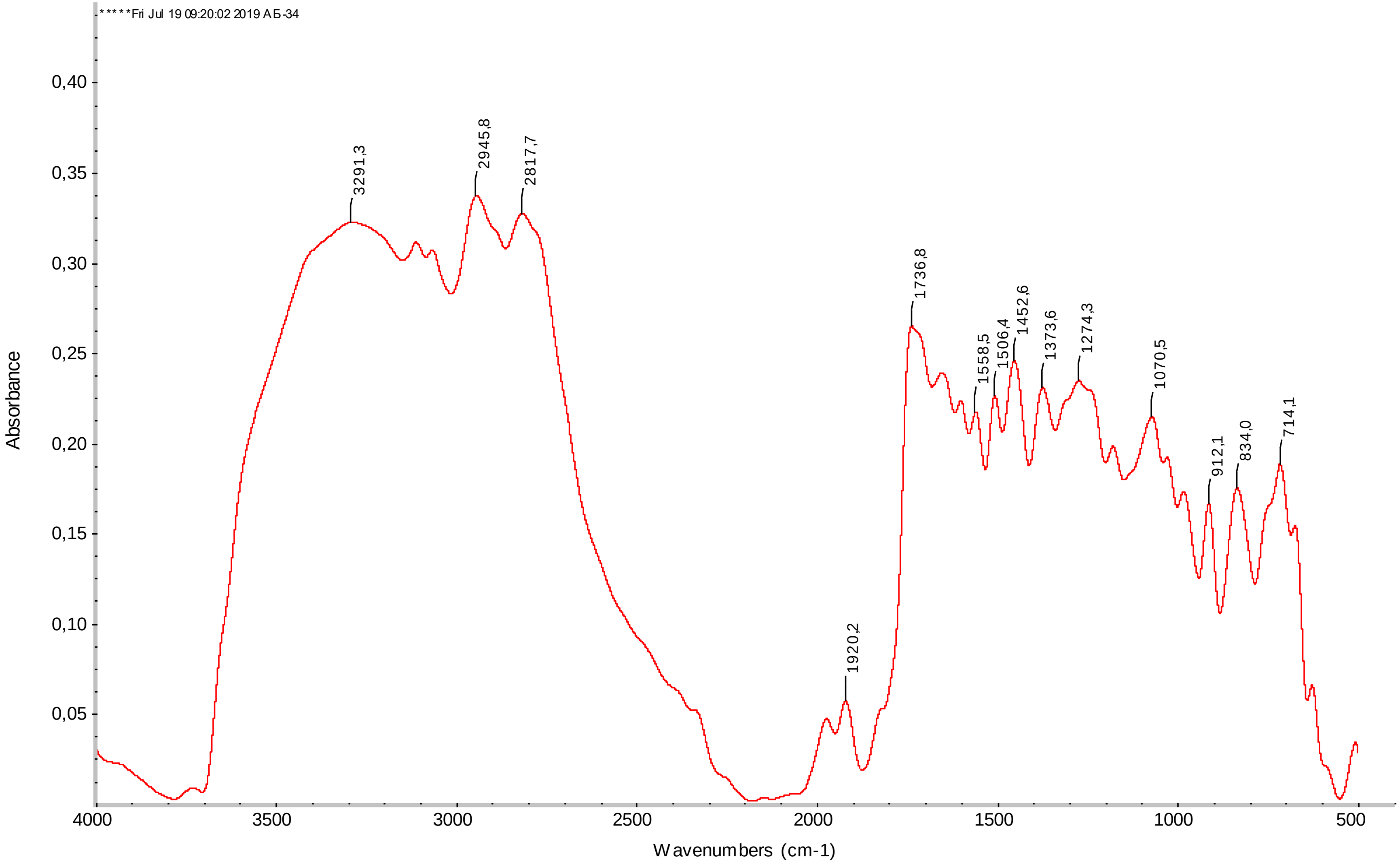

Figure S7. IR spectrum of O-benzoyl oxime of 3-(3-hydroxypropyl)-7-[3-(*1H*-imidazol-1-yl)propyl]-3,7-diazabicyclo[3.3.1]nonan-9-one (6b)

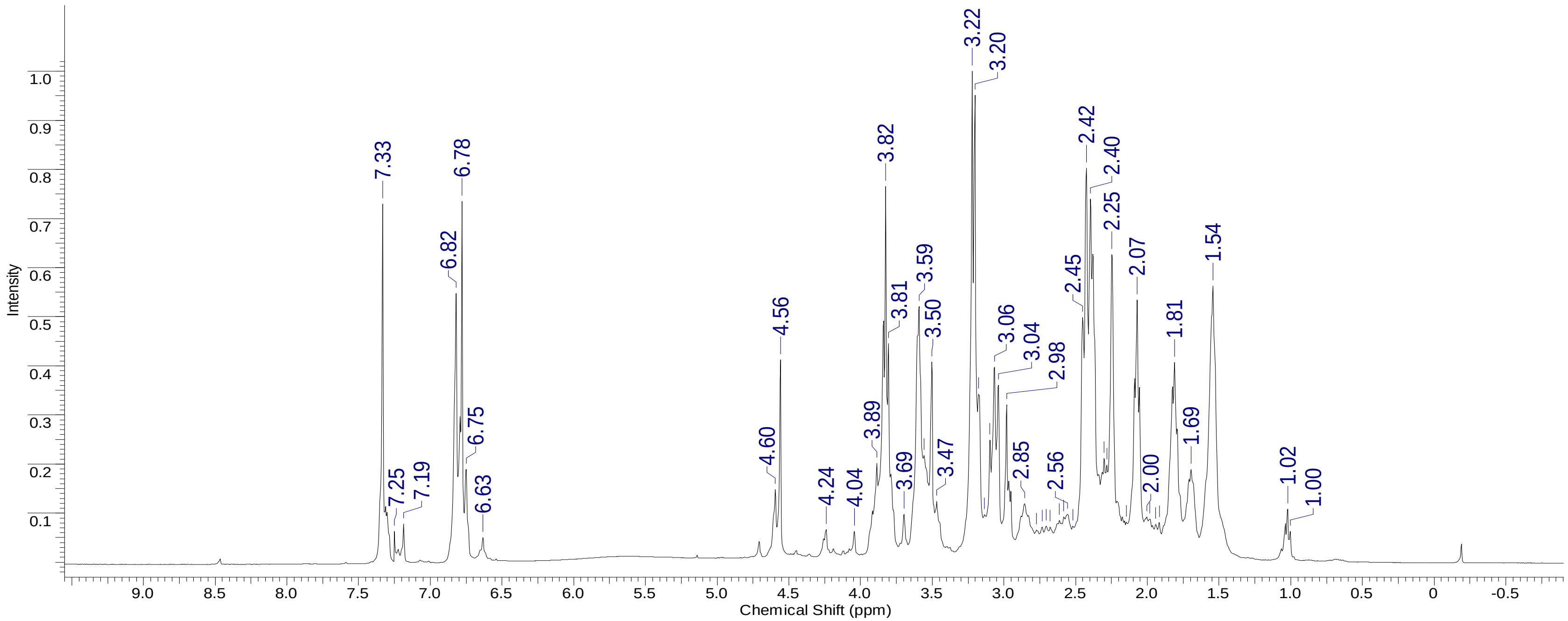

No.	(ppm)	(Hz)	Height	No.	(ppm)	(Hz)	Height	No.	(ppm)	(Hz)	Height	No.	(ppm)	(Hz)	Height
1	1.00	401.3	0.0629	15	2.40	958.3	0.7392	29	3.06	1225.3	0.3975	43	4.04	1615.9	0.0628
2	1.02	407.4	0.1084	16	2.42	968.9	0.8030	30	3.10	1239.0	0.2486	44	4.24	1695.3	0.0668
3	1.54	616.5	0.5625	17	2.45	979.6	0.4987	31	3.14	1254.3	0.0966	45	4.56	1821.9	0.4126
4	1.69	677.5	0.1893	18	2.52	1007.1	0.0736	32	3.18	1269.5	0.3421	46	4.60	1837.2	0.1484
5	1.81	723.3	0.4072	19	2.56	1022.3	0.0972	33	3.20	1280.2	0.9515	47	6.63	2652.0	0.0507
6	1.92	766.0	0.0810	20	2.58	1033.0	0.0919	34	3.22	1287.9	1.0000	48	6.75	2697.8	0.1895
7	1.94	776.7	0.0772	21	2.61	1045.2	0.0850	35	3.47	1387.0	0.1242	49	6.78	2710.0	0.7354
8	1.98	793.5	0.0875	22	2.68	1071.2	0.0714	36	3.50	1400.8	0.4083	50	6.82	2726.8	0.5472
9	2.00	801.1	0.0918	23	2.71	1081.9	0.0735	37	3.56	1422.1	0.2176	51	7.19	2873.2	0.0782
10	2.07	828.6	0.5338	24	2.73	1092.5	0.0717	38	3.59	1435.9	0.5212	52	7.25	2899.2	0.0632
11	2.15	857.6	0.0812	25	2.77	1109.3	0.0658	39	3.69	1477.1	0.0973	53	7.33	2931.2	0.7295
12	2.25	898.8	0.6265	26	2.85	1141.4	0.1193	40	3.81	1521.3	0.4456				
13	2.28	912.5	0.1977	27	2.98	1191.7	0.3205	41	3.82	1528.9	0.7653				
14	2.30	920.1	0.2114	28	3.04	1214.6	0.3607	42	3.89	1553.4	0.2020				

Figure S8. ^{1}H NMR spectrum of 1-(3-hydroxypropyl)piperidin-4-one (**1b)_**in_$CHCl_3$

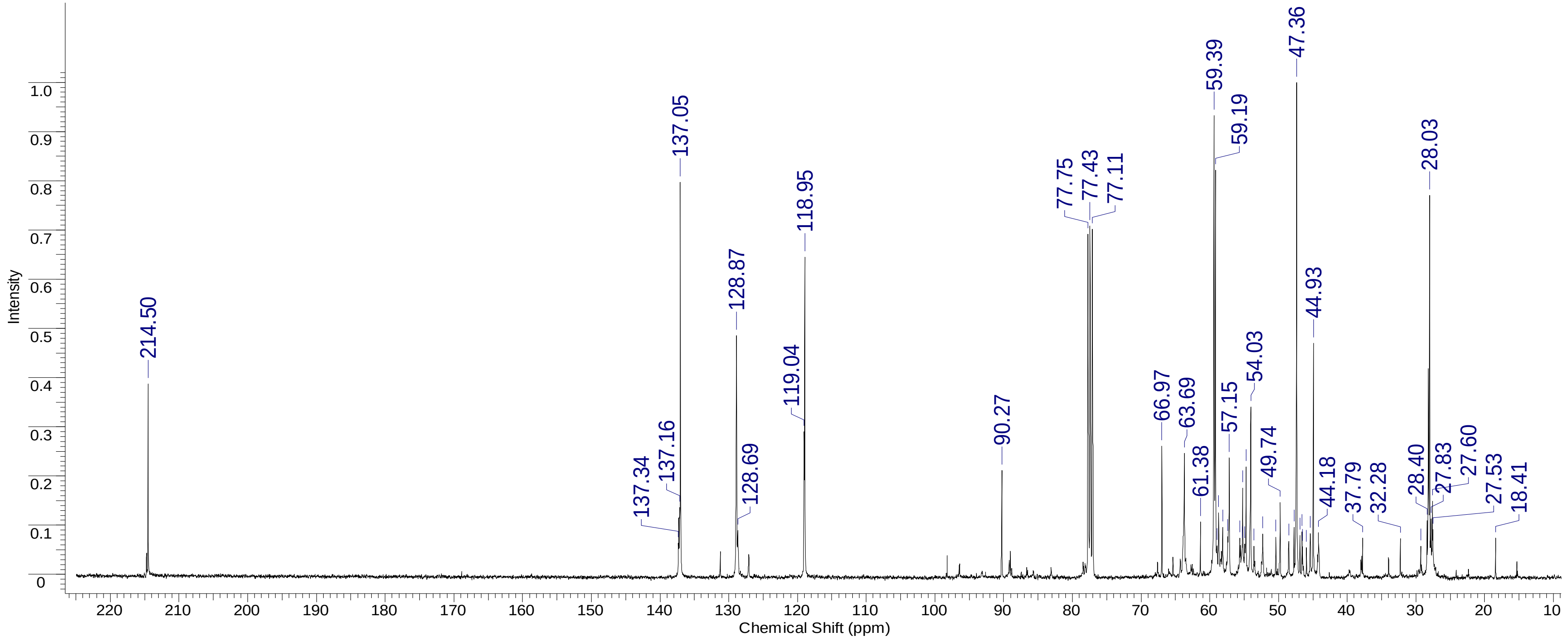

No.	(ppm)	(Hz)	Height	No.	(ppm)	(Hz)	Height	No.	(ppm)	(Hz)	Height	No.	(ppm)	(Hz)	Height
1	18.41	1850.9	0.0738	16	45.94	4618.0	0.0548	31	55.30	5559.2	0.0621	46	77.63	7804.0	0.3023
2	27.53	2767.2	0.0915	17	46.50	4674.6	0.0559	32	55.54	5583.2	0.0588	47	77.75	7815.5	0.6918
3	27.60	2774.9	0.1485	18	46.58	4682.2	0.0867	33	55.65	5593.7	0.0739	48	90.27	9073.9	0.2111
4	27.70	2784.5	0.0537	19	46.87	4712.0	0.0789	34	57.15	5745.2	0.2371	49	118.95	11957.0	0.6447
5	27.83	2797.9	0.1132	20	47.36	4760.8	1.0000	35	57.40	5770.1	0.0766	50	119.04	11966.6	0.2898
6	27.93	2807.5	0.0674	21	47.74	4799.2	0.0955	36	58.13	5843.9	0.0958	51	128.69	12936.6	0.0891
7	28.03	2818.0	0.7700	22	48.50	4875.9	0.0669	37	58.71	5901.4	0.1253	52	128.87	12954.8	0.4853
8	28.22	2837.2	0.4182	23	49.74	5000.5	0.1465	38	58.94	5925.4	0.0579	53	137.05	13777.1	0.7972
9	28.40	2854.4	0.1099	24	50.37	5063.7	0.0756	39	59.19	5950.3	0.8214	54	137.16	13787.7	0.1362
10	29.31	2946.5	0.0573	25	52.32	5259.2	0.0819	40	59.39	5970.4	0.9327	55	137.26	13798.2	0.1148
11	32.28	3244.5	0.0732	26	53.59	5386.7	0.0569	41	61.38	6169.8	0.1068	56	137.34	13805.9	0.0634
12	37.79	3798.5	0.0739	27	54.03	5431.8	0.3406	42	63.69	6402.7	0.2461	57	214.50	21562.8	0.3870
13	44.18	4440.7	0.0848	28	54.71	5499.8	0.2190	43	66.97	6732.4	0.2609				
14	44.93	4516.4	0.4697	29	54.94	5522.8	0.0615	44	77.11	7751.3	0.7021				
15	45.39	4562.4	0.0829	30	55.23	5551.6	0.1754	45	77.43	7783.8	0.7081				

Figure S9. ^{13}C NMR spectrum of 1-(3-hydroxypropyl)piperidin-4-one (**1b)**_in_$CHCl_3$

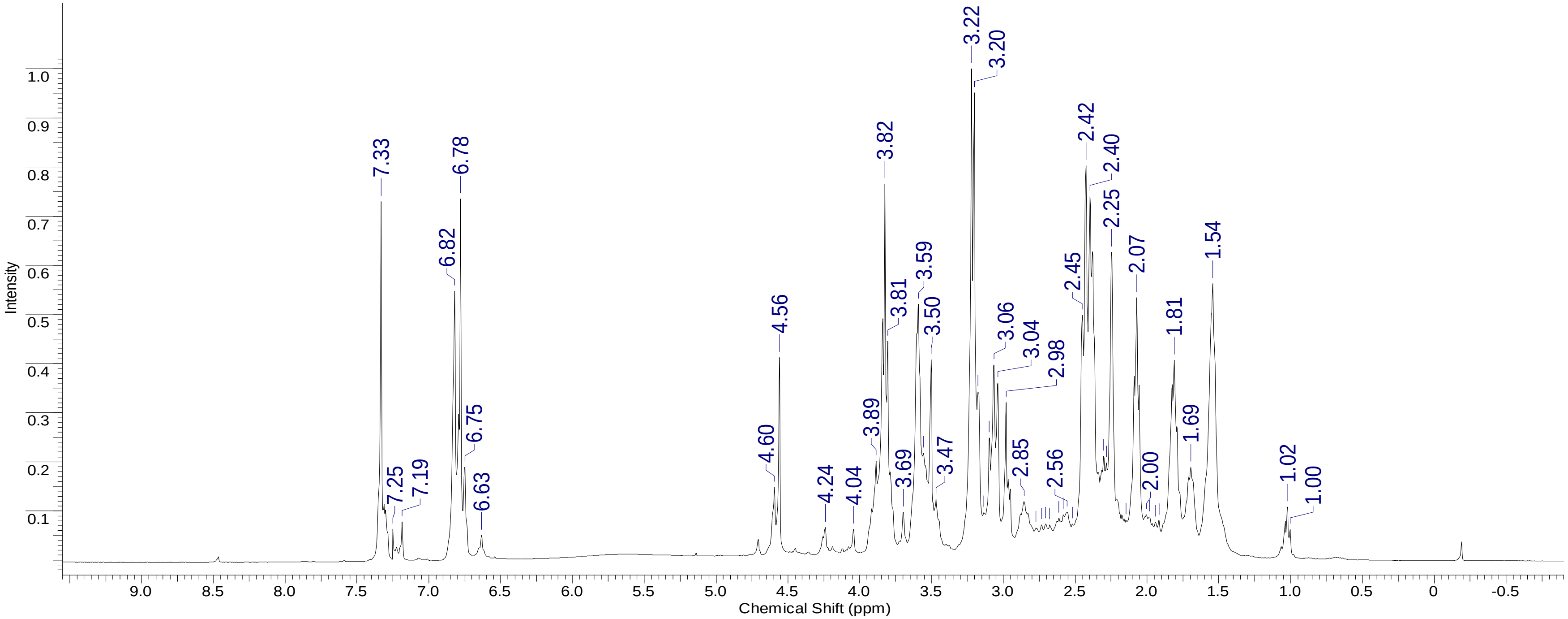

No.	(ppm)	(Hz)	Height
1	1.00	401.3	0.0629
2	1.02	407.4	0.1084
3	1.54	616.5	0.5625
4	1.69	677.5	0.1893
5	1.81	723.3	0.4072
6	1.92	766.0	0.0810
7	1.94	776.7	0.0772
8	1.98	793.5	0.0875
9	2.00	801.1	0.0918
10	2.07	828.6	0.5338
11	2.15	857.6	0.0812
12	2.25	898.8	0.6265
13	2.28	912.5	0.1977
14	2.30	920.1	0.2114

No.	(ppm)	(Hz)	Height
15	2.40	958.3	0.7392
16	2.42	968.9	0.8030
17	2.45	979.6	0.4987
18	2.52	1007.1	0.0736
19	2.56	1022.3	0.0972
20	2.58	1033.0	0.0919
21	2.61	1045.2	0.0850
22	2.68	1071.2	0.0714
23	2.71	1081.9	0.0735
24	2.73	1092.5	0.0717
25	2.77	1109.3	0.0658
26	2.85	1141.4	0.1193
27	2.98	1191.7	0.3205
28	3.04	1214.6	0.3607

No.	(ppm)	(Hz)	Height
29	3.06	1225.3	0.3975
30	3.10	1239.0	0.2486
31	3.14	1254.3	0.0966
32	3.18	1269.5	0.3421
33	3.20	1280.2	0.9515
34	3.22	1287.9	1.0000
35	3.47	1387.0	0.1242
36	3.50	1400.8	0.4083
37	3.56	1422.1	0.2176
38	3.59	1435.9	0.5212
39	3.69	1477.1	0.0973
40	3.81	1521.3	0.4456
41	3.82	1528.9	0.7653
42	3.89	1553.4	0.2020

No.	(ppm)	(Hz)	Height
43	4.04	1615.9	0.0628
44	4.24	1695.3	0.0668
45	4.56	1821.9	0.4126
46	4.60	1837.2	0.1484
47	6.63	2652.0	0.0507
48	6.75	2697.8	0.1895
49	6.78	2710.0	0.7354
50	6.82	2726.8	0.5472
51	7.19	2873.2	0.0782
52	7.25	2899.2	0.0632
53	7.33	2931.2	0.7295

Figure S10. ^{1}H NMR spectrum of 3-(3-hydroxypropyl)-7-[3-(1*H*-imidazol-1-yl)propyl]-3,7-diazabicyclo[3.3.1]nonan-9-one (**2b)**_in_$CHCl_3$

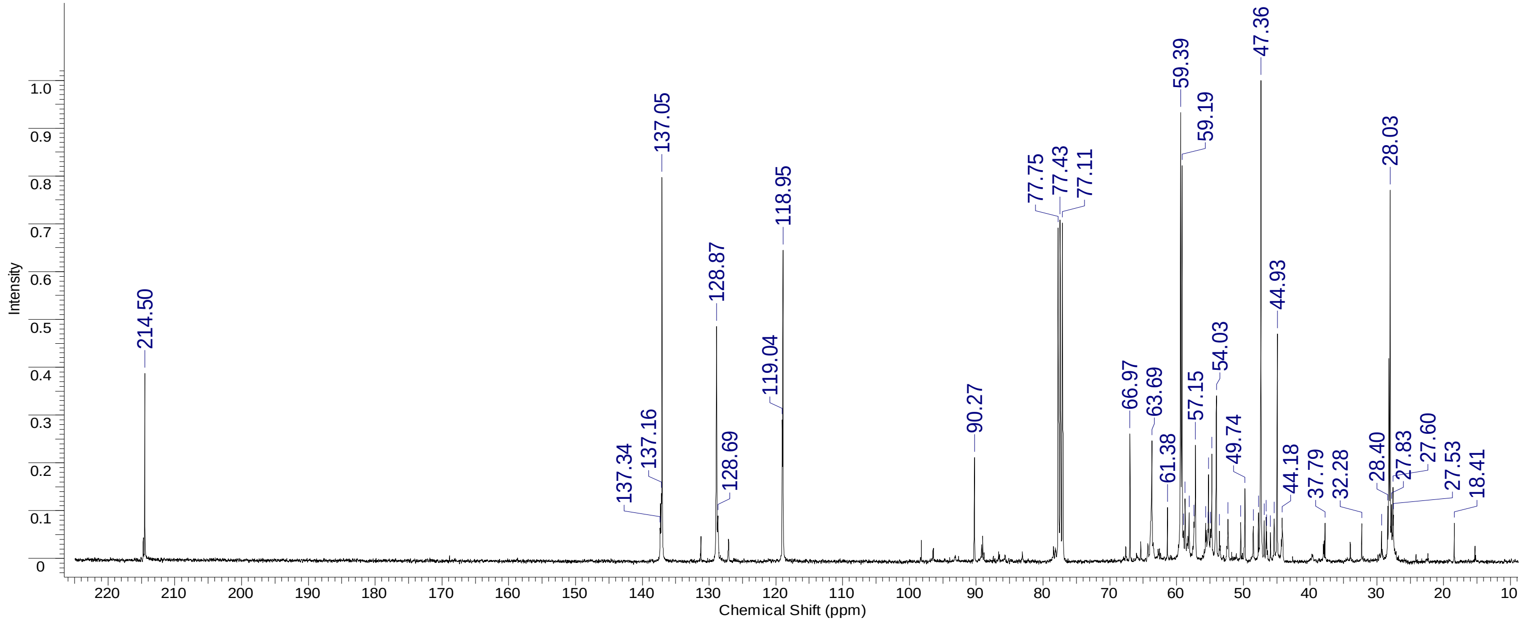

No.	(ppm)	(Hz)	Height
1	18.41	1850.9	0.0738
2	27.53	2767.2	0.0915
3	27.60	2774.9	0.1485
4	27.70	2784.5	0.0537
5	27.83	2797.9	0.1132
6	27.93	2807.5	0.0674
7	28.03	2818.0	0.7700
8	28.22	2837.2	0.4182
9	28.40	2854.4	0.1099
10	29.31	2946.5	0.0573
11	32.28	3244.5	0.0732
12	37.79	3798.5	0.0739
13	44.18	4440.7	0.0848
14	44.93	4516.4	0.4697
15	45.39	4562.4	0.0829
16	45.94	4618.0	0.0548
17	46.50	4674.6	0.0559
18	46.58	4682.2	0.0867
19	46.87	4712.0	0.0789
20	47.36	4760.8	1.0000
21	47.74	4799.2	0.0955
22	48.50	4875.9	0.0669
23	49.74	5000.5	0.1465
24	50.37	5063.7	0.0756
25	52.32	5259.2	0.0819
26	53.59	5386.7	0.0569
27	54.03	5431.8	0.3406
28	54.71	5499.8	0.2190
29	54.94	5522.8	0.0615
30	55.23	5551.6	0.1754
31	55.30	5559.2	0.0621
32	55.54	5583.2	0.0588
33	55.65	5593.7	0.0739
34	57.15	5745.2	0.2371
35	57.40	5770.1	0.0766
36	58.13	5843.9	0.0958
37	58.71	5901.4	0.1253
38	58.94	5925.4	0.0579
39	59.19	5950.3	0.8214
40	59.39	5970.4	0.9327
41	61.38	6169.8	0.1068
42	63.69	6402.7	0.2461
43	66.97	6732.4	0.2609
44	77.11	7751.3	0.7021
45	77.43	7783.8	0.7081
46	77.63	7804.0	0.3023
47	77.75	7815.5	0.6918
48	90.27	9073.9	0.2111
49	118.95	11957.0	0.6447
50	119.04	11966.6	0.2898
51	128.69	12936.6	0.0891
52	128.87	12954.8	0.4853
53	137.05	13777.1	0.7972
54	137.16	13787.7	0.1362
55	137.26	13798.2	0.1148
56	137.34	13805.9	0.0634
57	214.50	21562.8	0.3870

Figure S11. ^{13}C NMR spectrum of 3-(3-hydroxypropyl)-7-[3-(1*H*-imidazol-1-yl)propyl]-3,7-diazabicyclo[3.3.1]nonan-9-one (**2b)**_in_$CHCl_3$

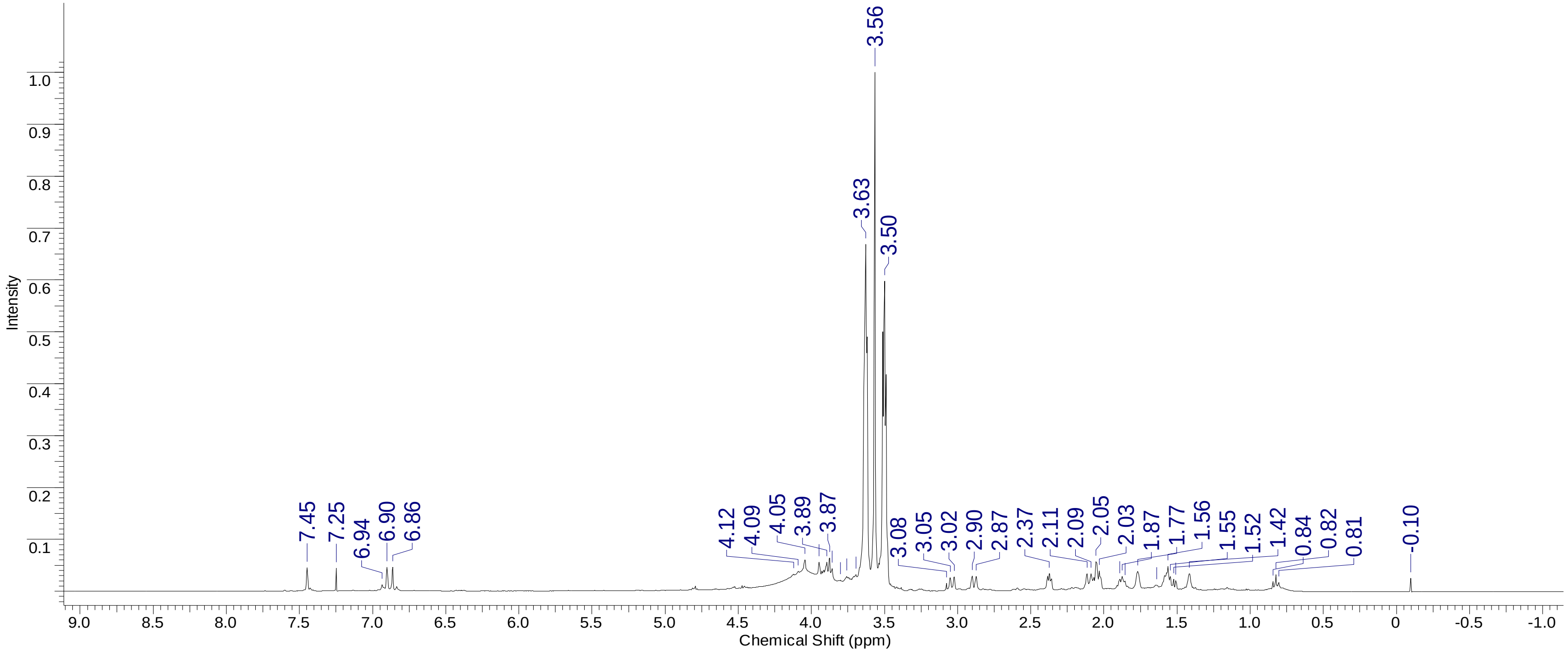

No.	(ppm)	(Hz)	Height
1	-0.10	-39.7	0.0254
2	0.81	322.0	0.0169
3	0.82	329.6	0.0317
4	0.84	337.2	0.0189
5	1.42	566.1	0.0334
6	1.51	602.7	0.0208
7	1.52	608.8	0.0234
8	1.55	618.0	0.0284
9	1.56	624.1	0.0478
10	1.64	654.6	0.0117
11	1.77	706.5	0.0378

No.	(ppm)	(Hz)	Height
12	1.85	741.6	0.0200
13	1.87	749.2	0.0289
14	1.89	756.8	0.0232
15	2.03	811.8	0.0389
16	2.05	820.9	0.0566
17	2.09	834.7	0.0333
18	2.11	845.3	0.0335
19	2.37	949.1	0.0339
20	2.87	1149.0	0.0287
21	2.90	1159.7	0.0297
22	3.02	1208.5	0.0277

No.	(ppm)	(Hz)	Height
23	3.05	1219.2	0.0260
24	3.08	1229.9	0.0155
25	3.50	1399.2	0.5973
26	3.56	1425.2	1.0000
27	3.63	1451.1	0.6684
28	3.69	1477.1	0.0324
29	3.76	1503.0	0.0281
30	3.80	1519.8	0.0213
31	3.86	1542.7	0.0438
32	3.87	1548.8	0.0637
33	3.89	1556.4	0.0561

No.	(ppm)	(Hz)	Height
34	3.95	1577.8	0.0556
35	4.05	1617.4	0.0602
36	4.09	1635.8	0.0385
37	4.12	1648.0	0.0326
38	6.86	2743.5	0.0458
39	6.90	2758.8	0.0462
40	6.94	2772.5	0.0128
41	7.25	2897.7	0.0442
42	7.45	2977.0	0.0452

Figure S12. ^{1}H NMR spectrum of 3-(3-hydroxypropyl)-7-[3-(1*H*-imidazol-1-yl)propyl]-3,7-diazabicyclo[3.3.1]nonane (**3b)**_in_$CHCl_3$

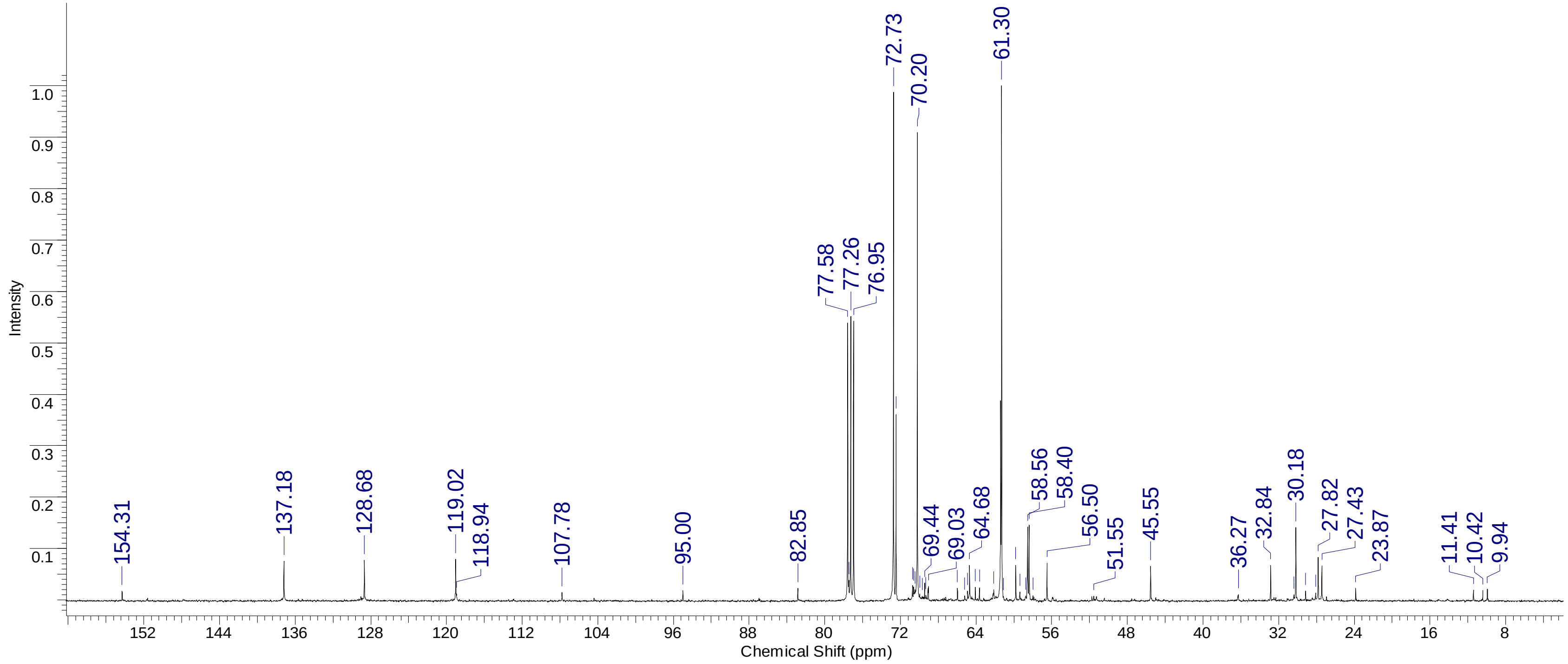

No.	(ppm)	(Hz)	Height	No.	(ppm)	(Hz)	Height	No.	(ppm)	(Hz)	Height	No.	(ppm)	(Hz)	Height
1	9.94	998.9	0.0216	16	56.50	5680.0	0.0718	31	64.89	6523.5	0.0179	46	76.95	7735.0	0.5423
2	10.42	1047.7	0.0183	17	57.98	5828.6	0.0089	32	65.20	6554.1	0.0086	47	77.26	7766.6	0.5518
3	11.41	1147.4	0.0198	18	58.40	5870.7	0.1460	33	65.97	6631.8	0.0235	48	77.46	7786.7	0.0385
4	23.87	2399.2	0.0231	19	58.56	5887.0	0.1426	34	69.03	6939.4	0.0264	49	77.58	7799.2	0.5388
5	27.43	2757.6	0.0668	20	58.73	5904.3	0.0086	35	69.08	6944.2	0.0230	50	82.85	8328.2	0.0231
6	27.82	2796.9	0.0834	21	59.37	5968.5	0.0160	36	69.31	6967.2	0.0091	51	95.00	9550.3	0.0184
7	28.11	2825.7	0.0138	22	59.80	6011.6	0.0681	37	69.44	6980.6	0.0328	52	107.78	10834.6	0.0148
8	29.16	2931.1	0.0179	23	61.09	6141.0	0.0073	38	69.70	7006.5	0.0079	53	118.94	11956.0	0.0118
9	30.18	3033.7	0.1410	24	61.30	6162.1	1.0000	39	69.95	7031.4	0.0124	54	119.02	11964.7	0.0791
10	30.40	3055.7	0.0104	25	61.40	6172.7	0.3879	40	70.20	7057.3	0.9083	55	128.68	12935.6	0.0773
11	32.84	3301.1	0.0678	26	62.14	6246.5	0.0206	41	70.45	7082.2	0.0201	56	137.18	13790.6	0.0760
12	36.27	3646.1	0.0116	27	62.20	6252.2	0.0132	42	70.61	7098.5	0.0257	57	154.31	15512.0	0.0180
13	36.33	3651.9	0.0098	28	63.63	6396.0	0.0236	43	70.73	7110.0	0.0288				
14	45.55	4578.7	0.0660	29	64.07	6441.0	0.0246	44	72.45	7283.5	0.3608				
15	51.55	5182.6	0.0080	30	64.68	6502.4	0.0674	45	72.73	7311.3	0.9870				

Figure S13. ^{13}C NMR spectrum of 3-(3-hydroxypropyl)-7-[3-(1*H*-imidazol-1-yl)propyl]-3,7-diazabicyclo[3.3.1]nonane (**3b)**_in_$CHCl_3$

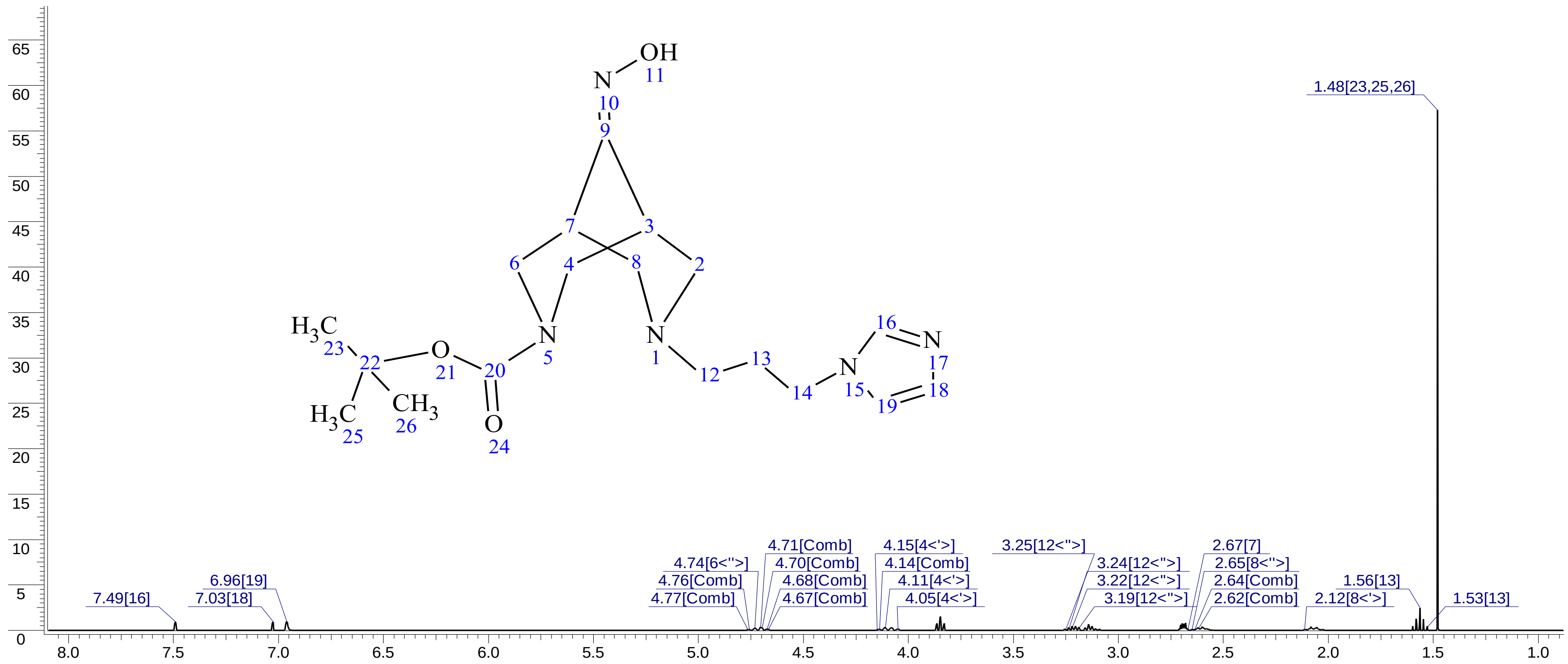

Group	nH	Shift	Error
2<">	1	2.60	0.34
2<'>	1	2.07	0.33
3	1	2.69	0.68
4<">	1	4.72	0.63
4<'>	1	4.10	0.40
6<">	1	4.72	0.63
6<'>	1	4.10	0.40
7	1	2.69	0.68
8<">	1	2.60	0.34
8<'>	1	2.07	0.33

Group	nH	Shift	Error
11	1	6.96	0.90
12<">	1	3.22	0.29
12<'>	1	3.13	0.26
13	2	1.56	0.13
14	2	3.85	0.21
16	1	7.49	0.11
18	1	7.03	0.09
19	1	6.96	0.15
23,25,26	9	1.48	0.05

Figure S14. ^{1}H NMR spectrum of oxime of 3-(3-Boc)-7-[3-(1*H*-imidazol-1-yl)propyl]-3,7-diazabicyclo[3.3.1]nonan-9-one (**5a)**_in_DMSO

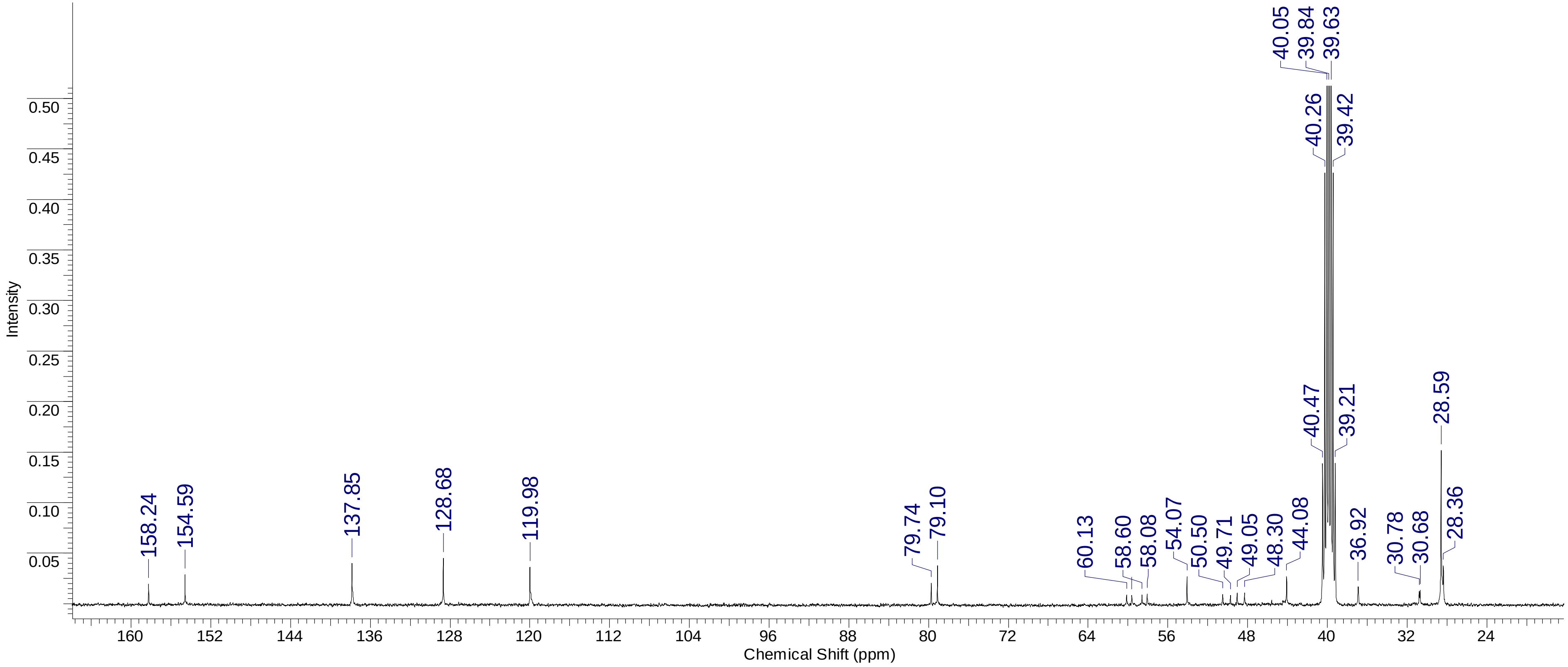

No.	(ppm)	(Hz)	Height
1	28.36	2850.6	0.0375
2	28.59	2873.6	0.1518
3	30.68	3084.5	0.0135
4	30.78	3094.1	0.0124
5	36.92	3711.3	0.0169
6	39.21	3941.3	0.1391
7	39.42	3962.4	0.4265
8	39.63	3983.5	0.8513
9	39.84	4004.6	1.0000
10	40.05	4025.7	0.8513

No.	(ppm)	(Hz)	Height
11	40.26	4046.8	0.4265
12	40.47	4067.9	0.1388
13	44.08	4431.1	0.0269
14	48.30	4855.7	0.0106
15	49.05	4930.5	0.0108
16	49.71	4997.6	0.0084
17	50.50	5076.2	0.0090
18	54.07	5435.6	0.0267
19	58.08	5838.2	0.0097
20	58.60	5890.9	0.0086

No.	(ppm)	(Hz)	Height
21	59.60	5991.5	0.0082
22	60.13	6044.2	0.0088
23	79.10	7951.6	0.0378
24	79.74	8015.8	0.0204
25	119.98	12061.5	0.0362
26	128.68	12935.6	0.0453
27	137.85	13857.6	0.0401
28	154.59	15539.8	0.0288
29	158.24	15906.8	0.0196

Figure S15. ^{13}C NMR spectrum of oxime of 3-(3-Boc)-7-[3-(1*H*-imidazol-1-yl)propyl]-3,7-diazabicyclo[3.3.1]nonan-9-one (**5a)**_in_DMSO

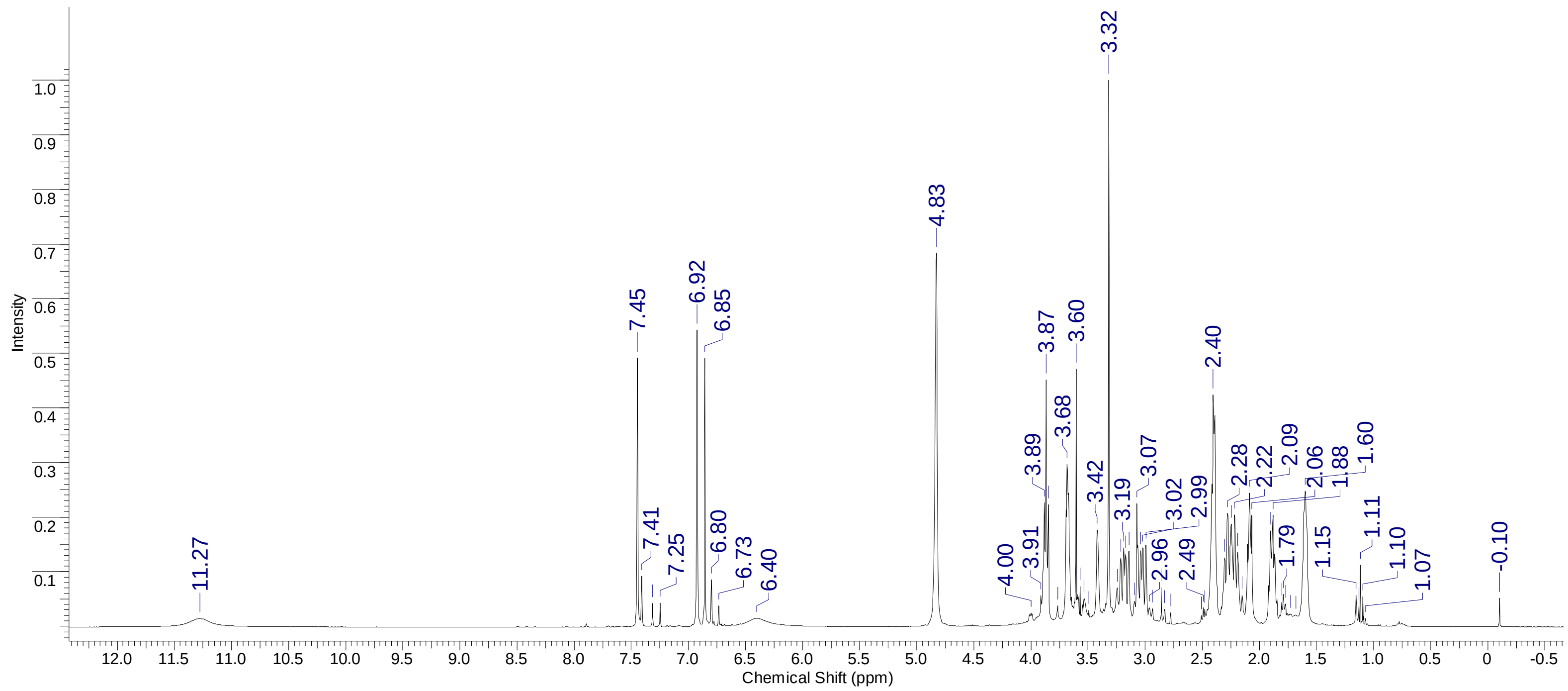

No.	(ppm)	(Hz)	Height
1	-0.10	-41.2	0.0513
2	1.07	428.8	0.0146
3	1.10	437.9	0.0547
4	1.11	445.6	0.1123
5	1.13	451.7	0.0362
6	1.15	460.8	0.0565
7	1.60	639.4	0.2477
8	1.68	671.4	0.0209
9	1.73	691.2	0.0228
10	1.77	708.0	0.0406
11	1.79	715.6	0.0588
12	1.81	721.8	0.0444
13	1.88	752.3	0.2029
14	1.90	759.9	0.1748
15	2.06	825.5	0.2029
16	2.09	834.7	0.2438
17	2.15	860.6	0.0573
18	2.19	875.9	0.1362
19	2.22	886.5	0.2042
20	2.24	897.2	0.1873
21	2.28	911.0	0.2066
22	2.31	921.6	0.1253
23	2.40	961.3	0.4237
24	2.48	990.3	0.0311
25	2.49	996.4	0.0336
26	2.51	1002.5	0.0200
27	2.77	1109.3	0.0253
28	2.83	1132.2	0.0311
29	2.86	1142.9	0.0714
30	2.94	1173.4	0.0321
31	2.96	1184.1	0.0335
32	2.99	1196.3	0.1489
33	3.02	1208.5	0.1435
34	3.04	1214.6	0.1384
35	3.07	1228.3	0.2248
36	3.10	1237.5	0.0456
37	3.14	1255.8	0.1377
38	3.17	1268.0	0.1311
39	3.19	1274.1	0.1438
40	3.21	1284.8	0.1253
41	3.24	1297.0	0.0703
42	3.32	1327.5	1.0000
43	3.42	1367.2	0.1763
44	3.49	1396.2	0.0298
45	3.53	1413.0	0.0504
46	3.57	1426.7	0.0730
47	3.60	1440.4	0.4707
48	3.68	1472.5	0.2965
49	3.77	1506.1	0.0370
50	3.85	1538.1	0.2225
51	3.87	1545.7	0.4513
52	3.89	1553.4	0.2263
53	3.91	1564.0	0.0559
54	4.00	1599.1	0.0239
55	4.83	1930.2	0.6824
56	6.40	2558.9	0.0146
57	6.73	2691.7	0.0377
58	6.80	2717.6	0.0857
59	6.85	2740.5	0.4908
60	6.92	2768.0	0.5424
61	7.25	2897.7	0.0426
62	7.31	2923.6	0.0420
63	7.41	2961.7	0.0917
64	7.45	2977.0	0.4913
65	11.27	4507.5	0.0142

Figure S16. ^{1}H NMR spectrum of oxime of 3-(3-hydroxypropyl)-7-[3-(1*H*-imidazol-1-yl)propyl]-3,7-diazabicyclo[3.3.1]nonan-9-one (5b**)**_in_$CHCl_3$

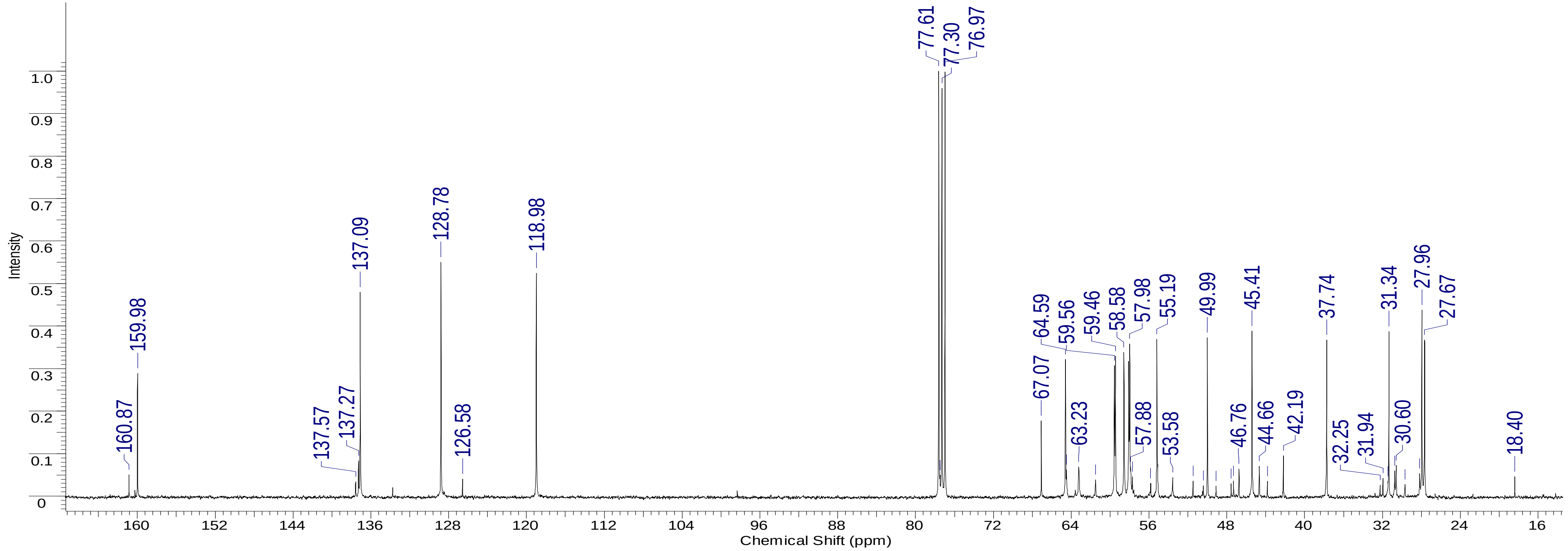

No.	(ppm)	(Hz)	Height	No.	(ppm)	(Hz)	Height	No.	(ppm)	(Hz)	Height	No.	(ppm)	(Hz)	Height
1	18.40	1850.0	0.0459	14	43.84	4407.2	0.0354	27	57.71	5801.7	0.0460	40	77.30	7770.4	0.9594
2	27.67	2781.6	0.3673	15	44.66	4489.6	0.0706	28	57.88	5818.0	0.0688	41	77.50	7790.5	0.0500
3	27.96	2810.4	0.4376	16	45.41	4565.3	0.3884	29	57.98	5828.6	0.3581	42	77.61	7802.1	1.0000
4	28.18	2832.4	0.0533	17	46.76	4700.5	0.0641	30	58.10	5840.1	0.3174	43	118.98	11960.8	0.5249
5	29.69	2984.8	0.0280	18	47.30	4755.1	0.0355	31	58.58	5889.0	0.3382	44	126.58	12724.7	0.0405
6	30.60	3075.9	0.0726	19	47.56	4781.0	0.0293	32	59.46	5977.1	0.3293	45	128.78	12946.1	0.5503
7	30.75	3091.2	0.0601	20	49.10	4936.2	0.0243	33	59.56	5987.7	0.3070	46	137.09	13781.0	0.4797
8	31.34	3150.6	0.3873	21	49.99	5025.4	0.3733	34	61.50	6182.2	0.0389	47	137.27	13799.2	0.0837
9	31.44	3160.2	0.0352	22	50.43	5069.5	0.0250	35	63.23	6356.7	0.0684	48	137.57	13828.9	0.0345
10	31.94	3211.0	0.0420	23	51.48	5174.9	0.0359	36	64.48	6482.2	0.0620	49	159.98	16082.2	0.2891
11	32.25	3241.7	0.0256	24	53.58	5385.8	0.0439	37	64.59	6492.8	0.3215	50	160.87	16171.4	0.0502
12	37.74	3793.7	0.3674	25	55.19	5547.7	0.3694	38	67.07	6742.0	0.1776				
13	42.19	4241.3	0.0949	26	55.84	5612.9	0.0309	39	76.97	7737.8	0.9982				

Figure S17. ^{13}C NMR spectrum of oxime of 3-(3-hydroxypropyl)-7-[3-(1*H*-imidazol-1-yl)propyl]-3,7-diazabicyclo[3.3.1]nonan-9-one (5b**)**_in_$CHCl_3$

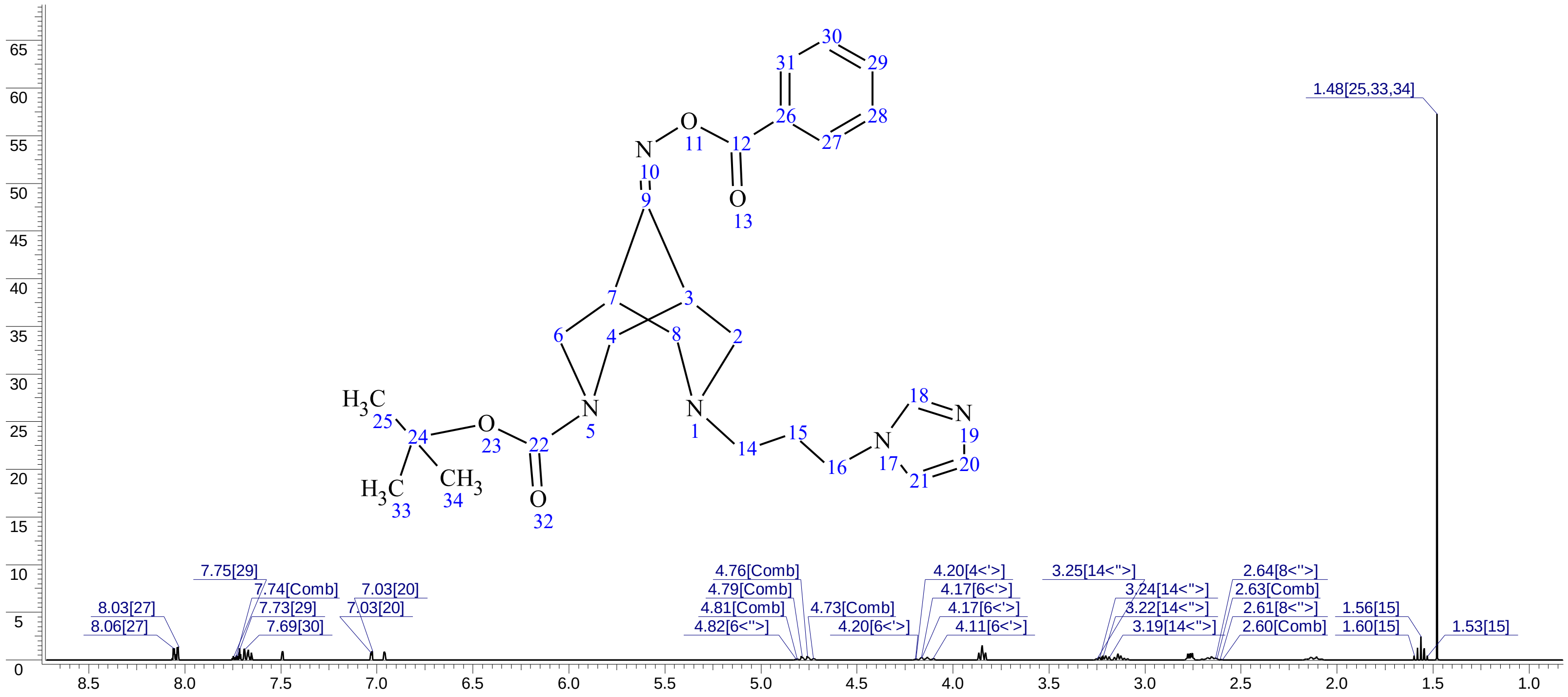

Group	nH	Shift	Error
2<">	1	2.65	0.34
2<'>	1	2.12	0.33
3	1	2.77	0.68
4<">	1	4.77	0.63
4<'>	1	4.15	0.40
6<">	1	4.77	0.63
6<'>	1	4.15	0.40
7	1	2.77	0.68
8<">	1	2.65	0.34
8<'>	1	2.12	0.33
14<">	1	3.22	0.29
14<'>	1	3.13	0.26

Group	nH	Shift	Error
15	2	1.56	0.13
16	2	3.85	0.21
18	1	7.49	0.11
20	1	7.03	0.09
21	1	6.96	0.15
25,33,34	9	1.48	0.05
27	1	8.05	0.03
28	1	7.67	0.01
29	1	7.73	0.03
30	1	7.67	0.01
31	1	8.05	0.03

Figure S18. ^{1}H NMR spectrum of O-benzoyloxime of 3-(3-Boc)-7-[3-(1*H*-imidazol-1-yl)propyl]-3,7-diazabicyclo[3.3.1]nonan-9-one (6a**)**_in_DMSO

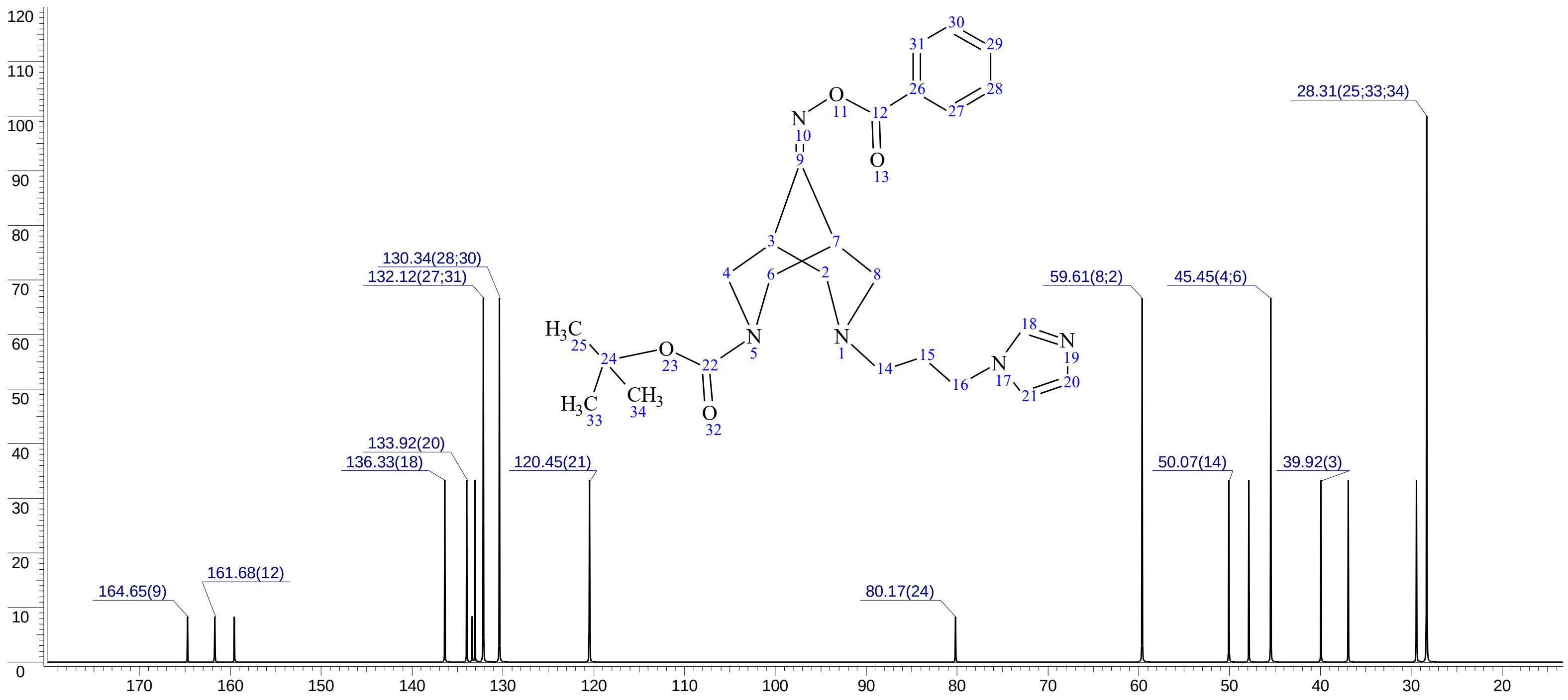

Carbon No.	CHn	Chem. Shifts	Conf. Limits
2	CH_2	59.61	2.4
3	CH	39.92	8.7
4	CH_2	45.45	5.3
6	CH_2	45.45	5.3
7	CH	36.92	8.7
8	CH_2	59.61	2.4
9	C	164.65	1.5
12	C	161.68	0.3
14	CH_2	50.07	4.3
15	CH_2	29.44	2.3
16	CH_2	47.85	4.7
18	CH	136.33	2
20	CH	133.92	1.5

Carbon No.	CHn	Chem. Shifts	Conf. Limits
21	CH	120.45	2
22	C	159.52	2.7
24	C	80.17	1
25	CH_3	28.31	1
26	C	133.35	---
27	CH	132.12	1.5
28	CH	130.34	0.7
29	CH	133	1.2
30	CH	130.34	0.7
31	CH	132.12	1.5
33	CH_3	28.31	1
34	CH_3	28.31	1

Figure S19. ^{13}C NMR spectrum O-benzoyloxime of 3-(3-Boc)-7-[3-(1*H*-imidazol-1-yl)propyl]-3,7-diazabicyclo[3.3.1]nonan-9-one (6a**)**_in_DMSO

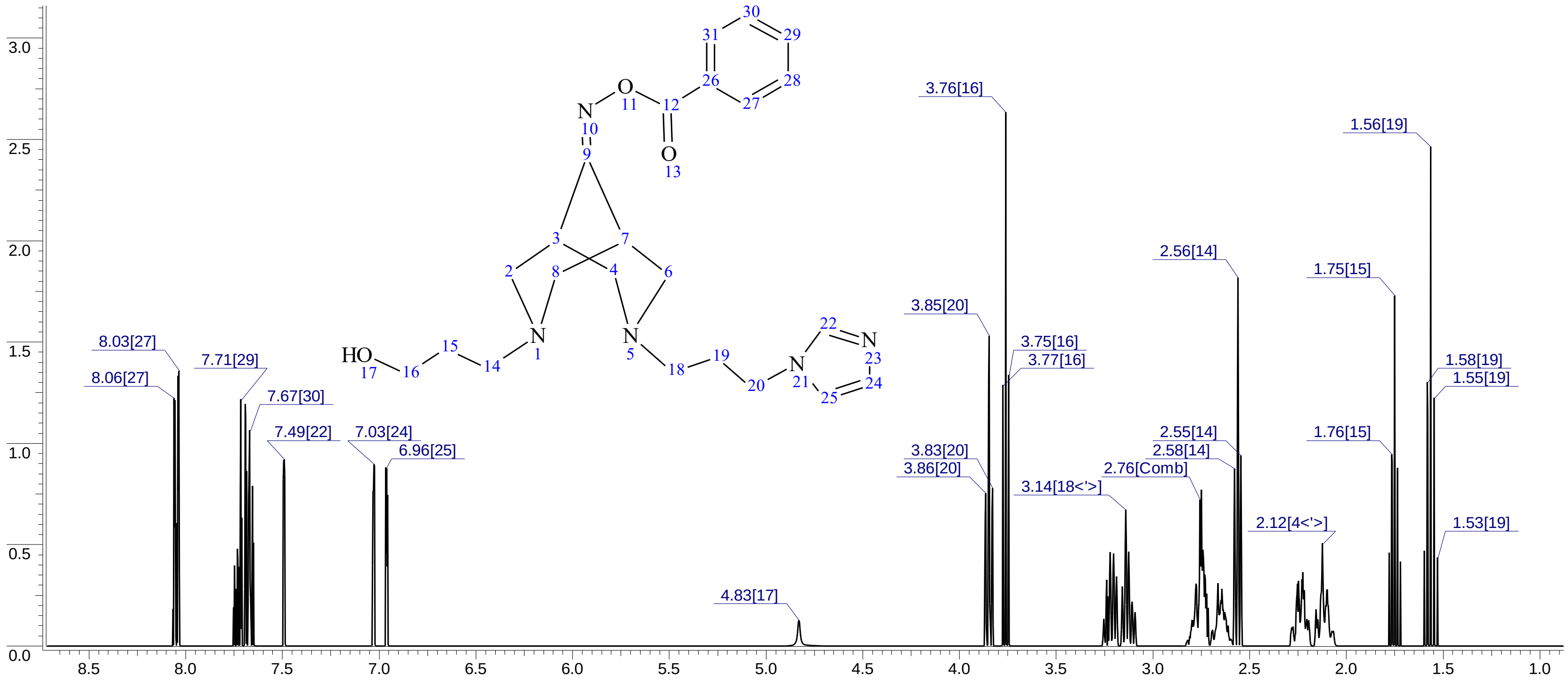

Group	nH	Shift	Error	Group	nH	Shift	Error
2<">	1	2.77	0.33	17	1	4.83	0.74
2<'>	1	2.24	0.33	18<">	1	3.22	0.29
3	1	2.74	0.68	18<'>	1	3.13	0.26
4<">	1	2.65	0.34	19	2	1.56	0.13
4<'>	1	2.11	0.33	20	2	3.85	0.21
6<">	1	2.65	0.34	22	1	7.49	0.11
6<'>	1	2.11	0.33	24	1	7.03	0.09
7	1	2.74	0.68	25	1	6.96	0.15
8<">	1	2.77	0.33	27	1	8.05	0.03
8<'>	1	2.24	0.33	28	1	7.67	0.01
14	2	2.56	0.07	29	1	7.73	0.03
15	2	1.75	0.05	30	1	7.67	0.01
16	2	3.76	0.08	31	1	8.05	0.03

Figure S20. ^{1}H NMR spectrum of O-benzoyloxime of 3-(3-hydroxypropyl)-7-[3-(1*H*-imidazol-1-yl)propyl]-3,7-diazabicyclo[3.3.1]nonan-9-one (6b**)**_in_CHCl$_3$

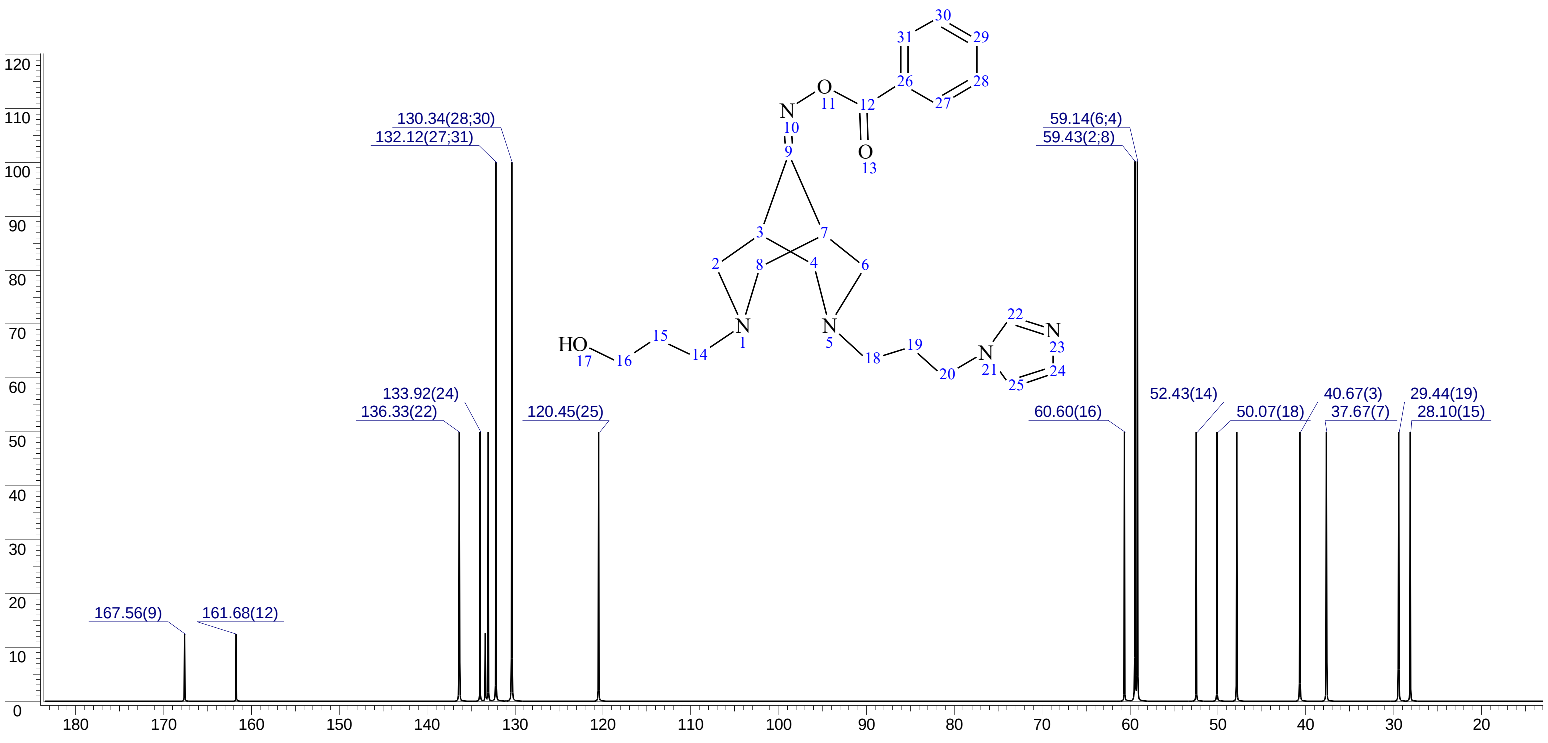

Carbon No.	CHn	Chem. Shifts	Conf. Limits
2	CH_2	59.43	2.5
3	CH	40.67	8.7
4	CH_2	59.14	2.5
6	CH_2	59.14	2.5
7	CH	37.67	8.7
8	CH_2	59.43	2.5
9	C	167.56	1.8
12	C	161.68	0.3
14	CH_2	52.43	2.2
15	CH_2	28.1	0.1
16	CH_2	60.6	0.2
18	CH_2	50.07	4.3

Carbon No.	CHn	Chem. Shifts	Conf. Limits
19	CH_2	29.44	2.3
20	CH_2	47.85	4.7
22	CH	136.33	2
24	CH	133.92	1.5
25	CH	120.45	2
26	C	133.35	---
27	CH	132.12	1.5
28	CH	130.34	0.7
29	CH	133	1.2
30	CH	130.34	0.7
31	CH	132.12	1.5

Figure S21. ^{13}C NMR spectrum of O-benzoyloxime of 3-(3-hydroxypropyl)-7-[3-(1*H*-imidazol-1-yl)propyl]-3,7-diazabicyclo[3.3.1]nonan-9-one (6b**)**_in_$CHCl_3$
